# Systematic Underestimation of Pesticide Burden for Invertebrates under Field Conditions: Comparing the Influence of Dietary Uptake and Aquatic Exposure Dynamics

**DOI:** 10.1021/acsenvironau.1c00023

**Published:** 2021-12-09

**Authors:** Benedikt B. Lauper, Eva Anthamatten, Johannes Raths, Maricor Arlos, Juliane Hollender

**Affiliations:** † Department of Environmental Chemistry, Eawag, 8600 Dübendorf, Switzerland; ‡ Institute of Biogeochemistry and Pollutant Dynamics, ETH Zürich, 8092 Zürich, Switzerland; § Department of Civil and Environmental Engineering, University of Alberta, 9211-116 St. NW, Edmonton, T6G 1H9 AB, Canada

**Keywords:** aquatic invertebrates, gammarids, pesticides, toxicokinetics, bioaccumulation, field study, dietary uptake, modeling

## Abstract

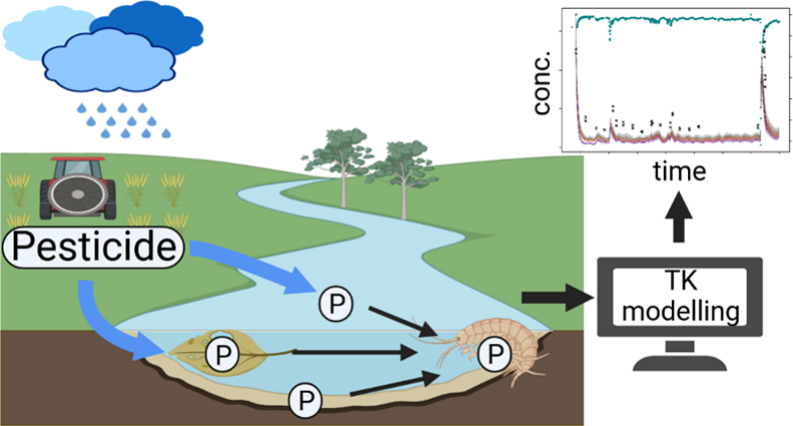

Pesticides used in agriculture can end up in nearby streams and can have a negative impact on nontarget organisms such as aquatic invertebrates. During registration, bioaccumulation potential is often investigated using laboratory tests only. Recent studies showed that the magnitude of bioaccumulation in the field substantially differs from laboratory conditions. To investigate this discrepancy, we conducted a field bioaccumulation study in a stream known to receive pollutant loadings from agriculture. Our work incorporates measurements of stream pesticide concentrations at high temporal resolution (every 20 min), as well as sediment, leaves, and caged gammarid analyses (every 2–24 h) over several weeks. Of 49 investigated pesticides, 14 were detected in gammarids with highly variable concentrations of up to 140 ± 28 ng/g_ww_. Toxicokinetic modeling using laboratory-derived uptake and depuration rate constants for azoxystrobin, cyprodinil, and fluopyram showed that despite the highly resolved water concentrations measured, the pesticide burden on gammarids remains underestimated by a factor of 1.9 ± 0.1 to 31 ± 3.0, with the highest underestimations occurring after rain events. Including dietary uptake from polluted detritus leaves and sediment in the model explained this underestimation only to a minor proportion. However, suspended solids analyzed during rain events had high pesticide concentrations, and uptake from them could partially explain the underestimation after rain events. Additional comparison between the measured and modeled data showed that the pesticide depuration in gammarids is slower in the field. This observation suggests that several unknown mechanisms may play a role, including lowered enzyme expression and mixture effects. Thus, it is important to conduct such retrospective risk assessments based on field investigations and adapt the registration accordingly.

## 1 Introduction

In the past decades, the number of chemicals used for personal, industrial, and agricultural purposes has increased significantly,^1^ with a high number of these compounds reaching water bodies via diffused pathways (e.g., runoff from agricultural fields) or point-source pollution (e.g., wastewater treatment plants).^2−5^ Many of the micropollutants (MPs) stemming from these sources are designed to be biologically active in target organisms (pesticides, pharmaceuticals); thus, they may affect nontarget aquatic organisms negatively^5−7^ due to the similarities in physiological functions (e.g., same or similar receptors and enzymes). To assess the full extent of MP exposure to aquatic organisms, it is important to understand how fast they are taken up and whether they bioaccumulate by the organisms of interest (toxicokinetics; TKs). Because uptake and elimination of MPs in organisms are complicated processes that differ not only between organisms but also among MPs, several endpoints such as bioconcentration factors (BCFs) or bioaccumulation factors (BAFs) have been used as surrogate measures. These parameters have been used in both research and environmental quality standard (EQS) derivation,^8−11^ with BCF as most commonly used for aquatic organisms. BCFs only consider the uptake of the chemical in the aqueous phase (either via respiratory or dermal exposure) and are usually determined in laboratory experiments where the target organisms are exposed to a constant MP concentration via the aqueous phase until they reach steady-state (uptake phase). Afterward, they are transferred into the uncontaminated aqueous medium where they are left to depurate (depuration phase).^11,12^ While this approach is simplistic, it is easily standardized and provides values that are comparable among MPs and organisms.^13^ By contrast, BAFs consider all possible routes of exposure including dietary uptake as well as uptake from ambient environmental sources such as contaminated sediment in the case of benthic invertebrates. BAFs are usually determined in the field or semi-field conditions such as mesocosms or flume channels to mimic natural environments^11,14^ where steady-state conditions are not always guaranteed.

Aquatic invertebrates, including gammarids, have been shown to be strongly impacted by pollution.^12,13,15^ Of the currently known MPs, pesticides have been shown to be the strongest driver for ecological risk in mixed-use watersheds.^16^ In addition to sensitivity of aquatic invertebrates to pesticide exposure, the TKs of small organisms such as gammarids are generally faster than those of larger organisms such as fish due to their higher surface to volume ratio.^17,18^ Hence, the faster TKs may lead to higher whole-body concentrations during short but intense exposure events such as agricultural runoff. As a result, it is likely that the severity of effects may be stronger in invertebrates compared to larger aquatic organisms. Because of their ubiquity in small streams, as well as their important ecological function as leaf shredders, gammarids have become common species for biomonitoring.^16,19−21^ There have been many studies that measure BCFs and the corresponding TKs of MPs in gammarids using laboratory trials.^22−24^ However, it has been shown that for some MPs, lab-derived BCFs are insufficient to predict the body burden in the field.^11,14,25^ For instance, Munz et al.^14^ showed that the neonicotinoid insecticides and azole fungicides were often detected in higher whole-body concentrations than predicted based on water concentrations alone. Additionally, Xie et al. showed that the bioaccumulation of pharmaceuticals in both invertebrates and fish from a Chinese lake exceeded both laboratory-derived BCF^26^ and BAFs predicted using the log *D*_ow_ of the pharmaceuticals.^27^ Arnot and Gobas showed in a review of published laboratory BCFs and field BAFs over all chemical classes that for most compounds, the BCF underestimates the environmental BAF by 1–2 orders of magnitude.^11^ This underestimation is problematic because many of those tests are used in risk assessment and for authorization.^8,10^

We developed two hypotheses associated with this underestimation. First, we hypothesize that in previous field studies, the main exposure event was missed due to insufficient time resolution of the water analysis, leading to the underestimation of the true exposure. Agricultural pesticides reach surface water mainly as runoff during rain events in short but intense peaks which are easily missed or underestimated even when using automated composite sampling.^28^ The second hypothesis is that in addition to uptake via water, alternate exposure paths such as dietary uptake or exposure to contaminated sediment influence the bioaccumulation. Contaminated leaf material could reach the streams directly from treated plants when senescent leaves are transported by wind or runoff. Alternatively, plants that use the streams as a water source could take up systemic pesticides and incorporate them into their leaves, which can reach the stream again during abscission. Traditionally, the uptake of polar compounds from diet is assumed to be minimal. For instance, Ashauer et al.^29^ suggested that the dietary pathway is only responsible for <1% of the total uptake for 10 out of 12 studied compounds, except for the compounds where they found that dietary exposure made up 10% of the total uptake. By contrast, recent studies by Englert et al.^30^ and Bundschuh et al.^15^ showed the increased effect of pesticides (neonicotinoid and pyrethroid insecticides, respectively) on invertebrates by a factor of up to 8 when exposed to pesticides via contaminated food and aqueous exposure. This indicates that the pesticides in the food source were taken up by the invertebrates and reached the target site. Contaminated sediment could be another important uptake pathway of MPs into invertebrates. It was shown that amphipods can take up more than 1 g of sediment per g of bodyweight (wet weight; ww) daily.^31^ While binding to sediments is usually considered more relevant for apolar compounds, it has been shown that also pesticides such as prochloraz and biocides such as propiconazole can be bound to sediments.^32^

In this study, we focused our efforts on pesticides due to the distinct peak exposure patterns mentioned above, providing us with ideal exposure conditions to test our first hypothesis. Many of the pesticides are also designed for quick uptake and distribution in plants to maintain its efficacy over the whole plant (systemicity). This contaminated plant material can subsequently serve as a food source for invertebrates. Hence, there is an opportunity to assess additional exposure pathways (second hypothesis). We conducted a field exposure study using caged gammarids in a small Swiss stream known for its pesticide load. The water concentration was measured at high temporal resolution (every 20 min). We further employed a single compartment TK model to assess the applicability of laboratory-derived TK data to complex field situations. The model further allowed us to compare the relative impact of intermittent exposure conditions and the contributions of each exposure pathway, aqueous, dietary, and sedimentary exposures to the overall contaminant uptake in the field.

## 2 Materials and Methods

### 2.1 Field Study Design

The field experiment of this work was designed to capture all hypothesized entry pathways of MPs into gammarids. A small Swiss stream known for its pesticide load^33^ located in the Swiss midland was selected (study stream). Using a fully automated mobile LC-HRMS/MS system (MS2Field^28^), the MP concentrations in the aqueous phase were measured every 20 min over 6 weeks (from May 27 to July 7, 2019). Further information about the aqueous phase measurements and the MP identification can be found in Section 2.5 and in the publications by Stravs et al.^28^ and la Cecilia et al.^34^ Because the stream does not have a native gammarid population, gammarids from a nearby stream in the same catchment (source stream) were collected, caged, and deployed in the study stream (Section 2.2). They were left to acclimate to the new environment for 1 week, during which they were fed with local leaves (Section 2.3). Afterward, one cage (corresponding to two laboratory samples) was collected daily for MP analysis (Section 2.4). During and after rain events, sampling frequency was increased for higher time resolution of expected input events and the following depuration period. Leaf and sediment samples were also collected at each gammarid sampling point (Section 2.3) to model the dietary and sedimentary uptake, respectively. A complete list of all samples can be found in the Supporting Information.

### 2.2 Collection and Identification of Gammarids

Gammarids for caging were sampled at the source stream using kick net sampling as described in standard protocols for benthic macroinvertebrate biomonitoring in Switzerland.^35,36^ The source stream contains a mix species community consisting of a majority of *Gammarus fossarum* (83%) and a minority of *Gammarus pulex* (17%) (total *n* = 77), which was determined according to Althermatt et al.^37^ During sampling, clearly juvenile, visibly pregnant and parasitically infected gammarids were sorted out and roughly 50 of the remaining individuals were randomly selected and put into each cage in order to get as representative population samples as possible. No differentiation was made between *G. fossarum* and *G. pulex* and the mixture population was used for all samples because Nyman et al. showed that the bioaccumulation of azole pesticides was comparable between the two.^38^ Polypropylene cages were built in-house with a volume of 1 L and the top and bottom cut out and replaced with stainless-steel mesh (0.5 mm mesh size) for continuous water exchange. They were transported to the study site and installed parallel to the waterflow within 1 h. The water temperature of the study stream varied between 12.4 and 19.9 °C with an average of 15.8 ± 1.8 °C. The temperature profile of the study stream can be found in the Supporting Information (Figure S1).

Because the source stream might also receive a small agricultural input, two gammarid sample sets were taken directly at the source stream. One set was frozen immediately at −20 °C to measure the background contamination at the time of collection (field control), and the other was caged and transported to the laboratory where it was kept in artificial pond water^39^ until the first sample from the study stream was taken (lab control) to ensure that the acclimation period was long enough to eliminate all pesticide residues from the source stream and all pesticides measured in the study stream originated from there. Due to a longer dry period between rain events, two additional batches of gammarids had to be collected, caged, and deployed. For both times, additional field and laboratory control samples were taken as well. Furthermore, gammarids from a pristine reference site near Zurich, Switzerland^14^ (Mönchaltdorfer Aa, Grüningen; 47.273908 N, 8.790048 E) were collected in the same way and used as matrix blank samples.

Once installed in the study stream, the gammarids were fed with local detritus leaves that were exchanged daily. At each sampling timepoint, one cage was removed from the river and the gammarids were then rinsed with NanoPure water (NPW) and frozen on-site at −20 °C. They were kept at −20 °C until analysis.

For samples where enough biomass was available after duplicate MP analysis (seven study- and two source stream samples distributed over the whole time period), aliquots of four gammarids were taken to determine the water and lipid content. Water content was determined gravimetrically by freeze drying. The lipid content was determined by a modified version of the protocol applied by Kretschmann et al.^40^ Shortly, the dried gammarids were extracted twice using a 4:5:5.5 (v/v/v) mixture of isopropanol, cyclohexane, and water, followed by separation of the organic phase containing the lipids. They were subsequently dried in the oven at 60 °C, and the lipid amount was determined gravimetrically.

### 2.3 Collection of Leaf and Sediment Samples

Leaves and sediments were sampled simultaneously to the gammarid samples. Detritus leaves and sediment from five randomly selected locations around the cages were collected and a mixed sample was created for both leaves and sediment. A part of the leaf sample was used as food for the following day and the rest was frozen at −20 °C until analysis. The sediment was collected from roughly the top 2 cm using a shovel and the mixed sample was then sieved with a mesh size of 2 mm, left to settle, and the pore water was decanted before freezing the sample at −20 °C. The sediment was collected from the top layer, as it can be a habitat for gammarids, and deeper sediment layers will likely have a different concentration profile.^41^

Additionally, the cages accumulated particulate matter, which had been suspended in the water during the two largest rain events (June 12 and July 7). This particulate matter was also sampled from multiple cages as described above, and the resulting samples are referenced as suspended solid samples in the following sections.

### 2.4 Sample Extraction

Gammarid samples were extracted and measured following a modified protocol by Munz et al.^14^ using QuEChERS extraction, followed by online solid-phase extraction (SPE) and analysis by liquid chromatography coupled to high-resolution tandem mass spectrometry with electron spray ionization (LC-ESI-HRMS/MS). The thawed gammarids were rinsed with NPW and blot-dried, and 400 mg aliquots were weighed into microcentrifuge tubes (roughly 16 individuals). Afterward, 50 μL of isotopically labeled internal standard (ISTD) solution (0.2 mg/L) was spiked, and the samples were refrigerated at 4 °C overnight. The remaining organic solvent was evaporated in a stream of nitrogen before adding 500 mg of 1 mm zirconia/glass beads (Carl Roth GmbH, Germany) as well as 500 μL of acetonitrile (ACN) and NPW each. They were then homogenized and extracted using a FastPrep homogenizer (MP Biomedicals, Switzerland) in two cycles at 6.5 m/s for 20 s each, cooling the samples on ice in between. Subsequently, they were centrifuged at 20,000 *g* and −10 °C for 6 min, followed by the removal of 800 μL of supernatant into new microcentrifuge tubes. QuEChERS salt (4:1 MgSO_4_/NaCl, 300 mg) was then added, and the samples were vortexed for 1 min, followed by another centrifugation step and removal of the ACN phase into a new tube. Another 500 μL of ACN was added to the initial tube containing the homogenate and the extraction was repeated. The two ACN extracts were pooled and stored at −20 °C overnight. The following morning, they were centrifuged again and the supernatant was transferred to a new tube to remove the solid which precipitated during the low-temperature storage. Subsequently, lipids were removed using two heptane extraction steps. Each time 500 μL of heptane was added, the samples were vortexed for 1 min and centrifuged again before removal of the heptane. Final ACN extracts were stored in 2 mL amber HPLC vials at −20 °C until analysis. Leaf samples were rinsed with NPW and freeze-dried. Because the freeze-dried leaf samples were not homogenized sufficiently by the homogenization method described above, they were milled twice for 15 s, at a frequency of 30 Hz using an electric milling device (Resch MM400, Verder Scientific) equipped with tungsten carbide cells and a milling ball. Afterward, 500 mg aliquots of leaf powder were weighed into microcentrifuge tubes and subsequently analyzed the same as described above for the gammarid samples.

Sediment samples were extracted and measured following the accelerated solvent extraction (ASE) protocol by Chiaia-Hernández et al.^32^ with the liquid–liquid extraction clean up step being replaced by online SPE. Briefly, 6 g of the freeze-dried sediment was weighed and spiked with 25 μL of 0.2 mg/L ISTD solution. ASE cells were prepared with, from bottom to top, a 16.2 mm cellulose filter (Dionex, Olten, Switzerland), 1 g of activated Florisil, a second cellulose filter, and the sediment sample homogeneously mixed with 500 mg of hydromatrix (diatomaceous earth for solvent channeling). The samples were then extracted in two cycles with an ASE 350 system (Dionex) at 80 °C using a 70:30 (% v/v) mixture of ethyl acetate and acetone. More details regarding the ASE can be found in the original publication.^32^ Extracts were then transferred into two 10 mL centrifuge vials and gently evaporated to a combined volume of 500 μL using an automated nitrogen blow down evaporator (TurboVap LV, Biotage LLC) at 40 °C. During evaporation, the vials were washed down twice using 1 mL of methanol to facilitate the dilution into NPW for the online SPE later. Samples were then transferred into 2 mL HPLC vials and stored at −20 °C until analysis. Suspended solid samples were extracted exactly as sediment samples, with the only exception that due to its lower density, only 3.5 g of suspended solids fit inside the ASE cells for extraction.

### 2.5 Chemical Analysis

For all measurements, 200 μL of organic extract was diluted by NPW up to a total volume of 20 mL for online SPE^24^ and LC-ESI-HRMS/MS on a hybrid quadrupole Orbitrap HR-MS/MS system: a Q-exactive Plus system was used for gammarid and leaf samples, while a Q-exactive system was used for the sediment samples (both Thermo Fisher Scientific). Chromatographic separation was achieved for all sample types using a C18 column (Atlantis T3, 5 μm, 3 × 150 mm, Waters) and a mobile phase gradient of a methanol/water mixture (both with 0.1% formic acid). For sediment samples, an additional gradient step of washing with 100% isopropanol was added because the ASE extraction was done with more apolar solvents and resulted in the carryover of apolar matrix components otherwise.^32^ For further details regarding the online SPE and the HPLC-HRMS/MS setup, see the Supporting Information.

Target screening for 49 pesticides was performed in all sample types. The target list was created using knowledge from MP contamination of the stream in previous years,^33^ MPs identified in the aqueous phase of the study stream during the real time monitoring using the MS2Field,^28^ as well as MPs defined as priority compounds due to concern of their environmental abundance or toxicity.^16^ All target compounds were quantified in the positive ionization mode.

Quantification was done using TraceFinder 4.1 (Thermo Fisher Scientific) via internal standard calibration in NPW for leaf and sediment samples. Due to very strong matrix effects, quantification of gammarid samples was done using matrix matched calibration. For this, gammarid samples from a pristine site (see Section 2.2) were extracted and 200 μL of matrix blank extracts were spiked to the calibration samples, matching the amount of matrix of the gammarid samples.

The aqueous samples were automatically processed in the MS2Field as described elsewhere.^28^ Shortly, every 20 min, stream water was pumped into the trailer, filtered (2 μm stainless steel disk, Collins 9150, TWP Inc.), and spiked with ISTD solution and/or quantification standards before being adjusted to a constant volume. This was followed by online SPE, chromatographic separation using a C18 LC-column (X-Bridge BEH, 3 μm, 2.1 × 50 mm), and ESI-HRMS/MS analysis by a Q-exactive HF system (Thermo Fisher Scientific).

### 2.6 TK Modeling

The whole-body concentration of each pesticide in gammarids was modeled individually using the aqueous, leaf, and sediment concentrations as input data. For the model, each matrix (gammarids, water, leaves, and sediment) was assumed to be a single homogeneous compartment and that the gammarids take up pesticides from them. Furthermore, first-order kinetics were assumed for uptake and depuration. The system can then be described using eq 1
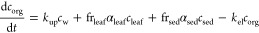
1where *c*_org_ is the whole-body concentration of the chemical in the gammarids, [ng/g_wet weight (ww)_], *c*_w_ [ng/L], *c*_leaf_ [ng/g_dry weight (dw)_], and *c*_sed_ [ng/g_dw_] are the chemical concentration in the water, leaves, and sediment, respectively. *k*_up_ is the kinetic rate constant of the aqueous uptake [L/kg·d] and *k*_el_ is the whole-body elimination rate of the gammarids [d^–1^]. For calculation, a unit conversion factor of 10^–3^ was applied to *k*_up_. fr_leaf_ [g_dw_/g_ww_·d] and fr_sed_ [g_dw_/g_ww_·d] are the daily feeding rates of the gammarids associated with leaves and sediment, respectively. Finally, α_leaf_ and α_sed_ are the assimilation coefficients from leaves and sediment and describe how much of the ingested pesticides are taken up into the gammarids. Because the pesticide concentration in the water, leaves, and sediment are not constant, no analytical solution for eq 1 exists. A python script was developed in SageMath 9.0^42^ where the differential equation is solved numerically using Heun’s method^43^ with 10,000 time-steps (Code in the Supporting Information). The model starts at the first sampling time and the average pesticide concentration of the timepoint is used as the starting concentration *c*_org_(*t* = 0) because the internal concentration at the start was pesticide specific. The model was then compared to measured internal concentrations to assist with our hypothesis testing and evaluation. In order to have sufficiently high time resolution, the input concentrations *c*_w_, *c*_leaf_, and *c*_sed_ were interpolated linearly also with 10,000 time-steps. If at a data point the pesticide was below the limit of quantification (LOQ), a concentration half of the LOQ was assumed. Because the last sampling point for leaves and sediment was a day before the end of the modeling period, the pesticide concentration in the leaves and sediment was assumed to remain constant at the last day.

In order to evaluate the influence of the different uptake pathways, the model was run using four different scenarios per pesticide. First, only respiratory uptake and elimination were considered. The second and third scenarios included only aqueous and dietary uptake from either leaves or sediment, while the last scenario considered all uptake pathways as described in eq 1. Upper and lower limits were calculated using error propagation from 95% confidence intervals (CIs), except for the feeding rates for which only standard deviations were available and they were not convertible to 95% CIs because no number of samples were given. This results in 95% CI error margins for the aqueous uptake and slightly smaller error margins than the 95% CIs for the versions including dietary and sedimentary uptake (further details in Section 6.1 of the Supporting Information).

Of the nine compounds that were detected above the LOQ for the majority of the exposure events (see Section 3.2), only one (azoxystrobin)^23,44^ had published TK rate constants (*k*_up_ and *k*_el_). Therefore, rate constants of four pesticides [azoxystrobin (AZO), cyprodinil (CYP), fluopyram (FLU), and thiacloprid (THI)] were determined in an accompanying study (see Section 5 in the Supporting Information for details on the laboratory experiment). For AZO, the newly measured values were used for modeling, to ensure consistency. When the literature data was used, the resulting modeled gammarid concentration lay within the model uncertainty (data not shown).

While there are studies that measured the feeding rates of gammarids, the values reported are very diverse and are affected not only by physical parameters such as temperature but also by chemical pollution^30,45,46^ and microbial cover.^47,48^ Because those parameters varied strongly over the whole test period and the pesticide concentrations found in both leaves and sediments were comparatively small, it was decided that for the modeling of the dietary uptake, a worst-case approach was warranted. Subsequently, the highest reported leaf-feeding rate of 0.43 ± 0.03 g_leaves dw_/g_gammarids dw_·d^45^ was used and adjusted to g_leaves dw_/g_gammarids ww_·d using the determined water content of the test gammarids. For the uptake of sediment, no value was reported for gammarids, but for *Hyalella azteca*, a value of 1.3 g_sediment dw_/g_gammarids ww_·d was reported.^31^ While gammarids are shredder organisms that feed on detritus and do not actively graze sediment as a food source, the detritus they eat is often covered by sediment particles that will most likely also be ingested during feeding. Thus, the effect of contaminated sediment will likely be lower for gammarids as for sediment grazing invertebrates, making this also viable for a worst-case approach. Because the analytical method for gammarids described above only allows for the determination of the whole-body concentration including chemicals absorbed to ingested material in the gut, both α_leaf_ and α_sed_ were set to 1 in order to assess the maximal possible uptake via diet. If the previously observed difference between laboratory and field gammarid concentrations were simply due to sorbed chemicals in the material in the gut, the model including dietary and sediment uptake with assimilation coefficients of 1 should explain the measured field data.

## 3 Results and Discussion

### 3.1 Water and Lipid Contents

The gammarid samples exposed to the study stream showed no significant difference in neither water nor lipid content compared to those from the source stream. The water content of study stream samples was measured to be 70 ± 2.8% (*n* = 14). The same was true for the lipid content with values of 4.4 ± 1.1%_ww_ (*n* = 14) for study stream samples. These values are in agreement with literature values.^12,14,22^

### 3.2 Target Screening

Relative recoveries of the pesticide analytes were between 70 and 130% in all three matrices, apart from two compounds in the gammarids, four in the leaves, and three in the sediment which showed higher deviations. Furthermore, three of the target compounds could not be quantified in gammarids and leaves (chlorpyrifos, chlorpyrifos-methyl, and pendimethalin). For a complete list of all recoveries see the Supporting Information.

In total, 14 pesticides (7 fungicides, 4 herbicides, and 3 insecticides) were detected above the LOQ in the gammarids over the whole sampling period. Of these, fungicides made up the highest proportion both by number of compounds and overall highest measured concentration [140 ± 28 ng/g_ww_ (*n* = 2) for FLU]. This fits to the land use of the catchment, where mainly fruits and berries are grown, which are often treated with fungicides.^49^ Additionally, four herbicides were detected with concentrations up to 47 ± 2.7 ng/g_ww_ for napropamide. Insecticides were detected the least in both number and concentration but THI was the most consistently detected compound detected at each timepoint with concentrations between 21 ± 3.7 and 44.0 ± 0.34 ng/g_ww_. Only two pesticides were detected in the laboratory control samples (CYP and THI). For the other twelve pesticides, it could be concluded that the total amount measured in the study stream samples originated from the study stream and were not attributed to the source stream. CYP was detected slightly above its LOQ with a concentration of 0.92 ± 0.52 ng/g_ww_ in the laboratory control samples, indicating that a small portion of the measured study stream concentrations were due to the pollution at the source stream. Because the values were low compared to the measured starting concentrations in the study stream (31 ± 11 ng/g_ww_) and because the model used the measured gammarid concentrations as starting values, no correction was applied to the study stream data. For a discussion regarding THI, see Section 3.4.1. In the water, 18 pesticides were detected: 9 fungicides, 6 herbicides, and 3 insecticides with peak concentrations up to 31 ± 6.2 μg/L (FLU). All pesticides detected in gammarids were also found in the water, with the exception of three compounds that were detected in the gammarids only barely above the LOQ (bupirimate, imidacloprid, and thiamethoxam). In the leaves, 31 pesticides (13 fungicides, 11 herbicides, and 6 insecticides) were detected with concentrations up to 840 ± 24 ng/g_dw_ for FLU. All pesticides detected in gammarids and the water were also detected in the leaves and the sediment. In the sediment, 37 pesticides (17 fungicides, 18 herbicides, and 10 insecticides) were detected.

The different number of pesticides detected in the various compartments matched the different sensitivities of the analytical methods applied. Pesticides detected in one compartment but not in the other were generally close to the LOQ in the detected compartment. The most number of compounds were detected in the sediment where the highest sample mass (6 g) was extracted using the ASE method resulting in LOQs in the pg/g_dw_ range. While the extraction method for the leaves and gammarids was the same, more total leaf mass was extracted, resulting in LOQs in the leaf samples mostly below 1 ng/g_dw_. Furthermore, a strong matrix effect was observed in the gammarid samples, thus reducing the sensitivity of the method which resulted in LOQs in the low ng/g_dw_ range (roughly one order of magnitude above the leaf method). The MS2Field method has LOQs between 5 and 70 ng/L.^28,34^ A full list of all pesticides screened, their LOQs, as well as maximal and minimal detected concentrations can be found in the Supporting Information.

### 3.3 Pesticide Concentration Dynamics

**Figure 1:**
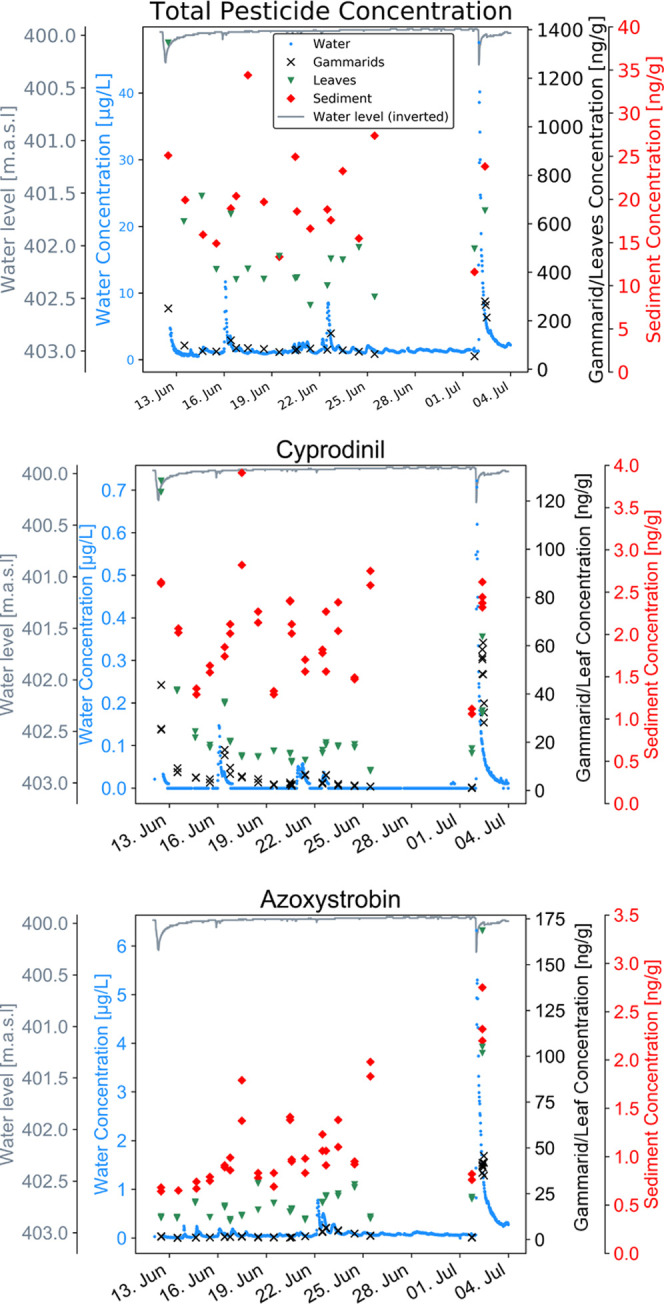
Time profile of the measured total pesticide concentration (top) in water, gammarids, leaves, and sediment and the profiles of cyprodinil (middle) and azoxystobin (bottom). Note that during the apex of the first rain event (12 June), no water concentrations could be measured because the pump of the MS2Field was clogged. The water level of the study stream was plotted as meter above sea level (m.a.s.l.) with inversed *y*-axis.

The water concentrations of all pesticides correlated with the water level in the study stream (Figure 1, Pearson’s *r* > 0.5, *p*-value < 0.05), showing that the pesticide concentrations increased linearly with the precipitation. This indicates that agricultural runoff during rain events was the main input pathway into the system because otherwise rain events would dilute the concentrations in the stream. AZO showed the lowest correlation (Pearson’s *r* = 0.52, *p*-value < 0.05) because its water concentration remained low during the first rain event. It was likely applied to the field only after the rain event, thus explaining this behavior. Furthermore, the time profiles of the gammarid concentrations show a strong correlation to the water concentration for all pesticides except THI (Pearson’s *r* > 0.9, *p*-values < 0.05). For THI discussion, see Section 3.4.1. The correlation of the leaves and sediment concentrations to the water concentration is strongly compound-specific ranging from strongly correlating (AZO in leaves: Pearson’s *r* = 0.94, *p*-value = 1.92 × 10^–9^) to very poor correlation (CYP in sediment: Pearson’s *r* = 0.19, *p*-value = 0.44). This is not surprising because gammarids actively respire the water, while the leaves and sediment come only passively into contact with the pesticides in the water resulting in uptake that is mostly governed by sorption, for which compound-specific parameters such as the *K*_OW_ and speciation have a stronger influence.

### 3.4 TK Modeling

Of the 11 compounds detected in all compartments, two (metamitron and terbutylazine) were only detected above the LOQ in a small number of peak exposure timepoints, making them unsuited for modeling due to the high uncertainty of pesticide concentration below the LOQ. This resulted in nine pesticides found in all compartments deemed enough for modeling. For four (AZO, CYP, FLU, and THI) of the nine remaining pesticides, TK rate constants were available and are thus discussed below. These four made up the largest part of the total pesticide burden (83%) in gammarids and contained both an azole fungicide (AZO) and a neonicotinoid insecticide (THI) for which field overestimation had been observed previously. Because of this, the subsequent discussion will focus on the results of these four compounds. Modeling results for the remaining five compounds show similar time trends as AZO, CYP, and FLU. They can be found in the Supporting Information.

#### 3.4.1 Aqueous Uptake

**Figure 2:**
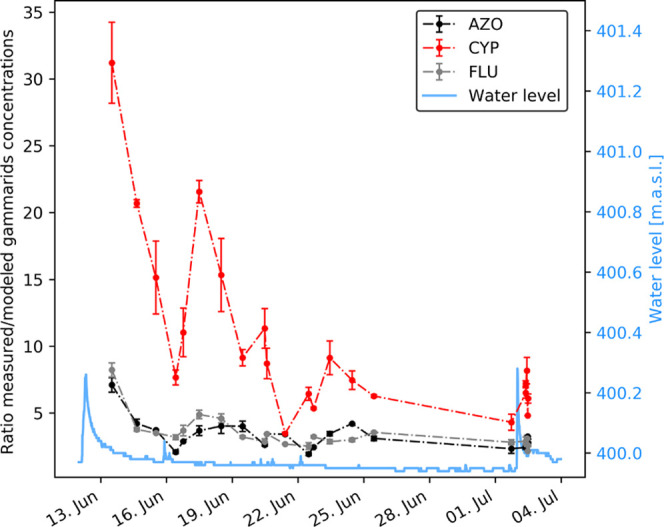
Factor by which the TK model underestimates the pesticide concentration in filed-exposed gammarids. Water level is presented as meter above sea level (m.a.s.l.). No data are shown for June 12 because then the measured concentrations were used as starting concentrations for the model. For measured pesticide concentrations at the timepoints see Figure 3.

**Figure 3:**
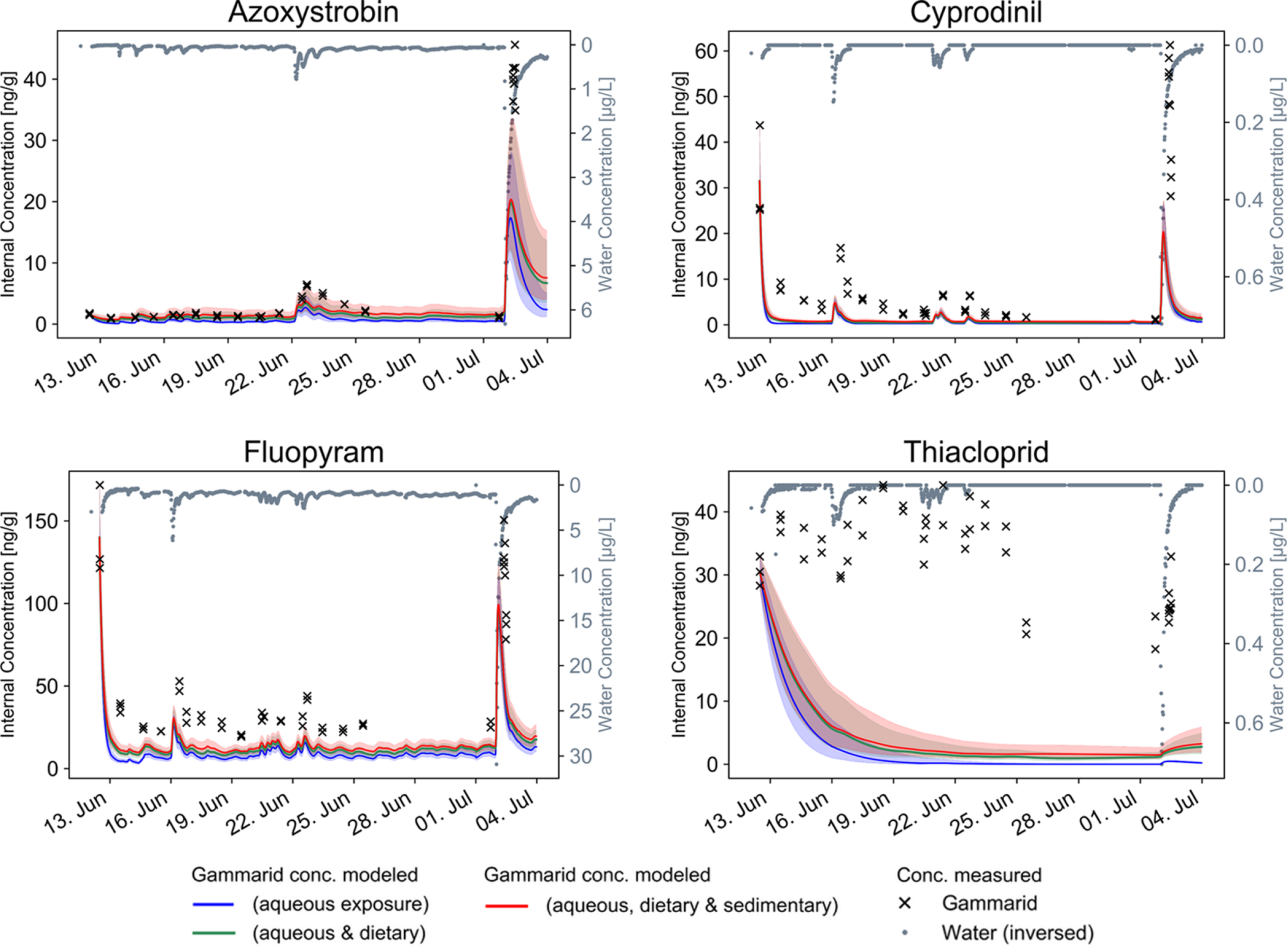
Modeled internal concentration of the pesticides AZO, CYP, FLU, and THI in gammarids when considering only aqueous uptake (blue line), aqueous and dietary uptake combined (green), and when considering uptake from sediment as well (red). The colored band represents the error range of the model (mainly 95% CIs; more information in Section 2.6). Their measured internal concentrations (black crosses) and the measured water concentrations (gray dots) are plotted for comparison.

A comparison of the modeled pesticide internal concentrations to the measured values (Figure 3) indicates that there is a good fit of the temporal trends between predicted and observed data. It was found that for AZO, CYP, and FLU, the modeled temporal trends match the measured data, but the pollution was underestimated over the whole time period. This agrees with the existing literature that compares accumulation of pesticides from the laboratory to the field,^11,14,26,27^ where a similar increase in the field was observed based on grab samples. The highest deviations were observed shortly after rain events (Figure 2), when the water concentrations were again low but the measured whole-body burden in gammarids were still elevated contrary to TK model prediction (see CYP and FLU on June 14 in Figure 3). This supports our first hypothesis that temporal variations need to be considered when calculating BAFs from field data. Most likely, the highest previously reported deviations^11,14,26,27^ resulted from data gathered after exposure events and would be smaller if temporal variations had been considered during sampling. While a high temporal resolution could reduce the deviation between laboratory and field-derived data, it does not explain it fully. FLU, for example, was predicted by the model to show rapid elimination from the initial measured concentration down to steady-state concentration within less than 24 h (Figure 3). However, the measured values were markedly slower, indicating slower depuration in the field compared to the lab. Similarly, measured values reached the modeled steady-state concentrations for CYP only after a week with low and very constant exposure on July 1, a lot slower as would be expected based on the TK rates. For FLU, the modeled steady-state concentration was not reached. Because the time to reach the steady state is dependent on the depuration rate constant, this further adds credence to the hypothesis that the depuration in the field is slow compared to the lab. A reduction of *k*_el_ by a factor of 3 to 4 would result in a fit for most data points but there is no scientific basis to support this approach (data not shown). Recently, Švara et al. proposed reduced depuration kinetics of imidacloprid in gammarids from polluted sites compared to gammarids from unpolluted sites due to changes in metabolic activity depending on pollution levels.^50^ Potentially, enzymes which metabolize the pesticides are expressed less in the field because the true pesticide pollution exposure concentrations were lower than what were used during laboratory trials (μg/L range). Hence, the induction of enzymes responsible for detoxification may have been lower as was shown previously for fish.^51−53^ Also, interactive effects of pesticide mixtures could reduce elimination efficiency, such as through inhibition of cytochrome P450 monooxygenases by azole fungicides^54,55^ or by pesticide synergists such as piperonyl butoxide which is a pesticide synergist commonly used in combination with pyrethrins or pyrethroids, which inhibits cytochrome P450 monooxygenases.

Finally, the difference in water temperature between the laboratory and the field study could influence the enzyme activity and subsequently the elimination rates. Because the water temperature in the field (12.4–19.9 °C; see Figure S1 in the Supporting Information for more information) was always higher than in the laboratory experiment (11 °C) and enzyme activity generally increases with the increase in temperature,^56^ one would expect higher enzyme activity and faster depuration kinetics in the field. However, the opposite was observed and subsequently, it is unlikely that the temperature difference between the laboratory and the field was responsible for the observed underestimation.

For THI, complete elimination would be expected based on the TK data and the measured concentrations in the other compartments. Contrary to this, a constant high pollution was measured with no clear indications of first-order depuration. Furthermore, THI was the only pesticide which was detected in the lab control samples (20 ± 3.1 ng/g_ww_, *n* = 6) at the same concentration as in the source stream samples (20 ± 1.7 ng/g_ww_, *n* = 6), further suggesting its resistance to depuration by the gammarids. Accordingly, a switch to a new batch of gammarids with lower concentrations from the source stream explains the concentration drop on June 25 down to concentrations comparable to the source stream samples (22 ± 1.3 ng/g_ww_, *n* = 2). The initial batch which was exposed to the large rain event of June 6 and samples exposed to the rain event on July 2 had an increased concentration up to 44 ± 0.34 ng/g_ww_ (*n* = 2) on June 18 after the second rain event, indicating that while it is not depurated, additional uptake does take place. THI has been shown to form complexes with metal ions such as Ag^2+^ and Cu^2+^.^57,58^ A similar binding to the Ca^2+^ in the chitinous exoskeleton^59^ or in another compartment would make THI difficult to excrete and inaccessible for biotransformation enzymes. Investigations regarding the binding of THI to the exoskeleton using MS-imaging are ongoing.

#### 3.4.2 Uptake via Diet and Sediment Exposure

**Figure 4:**
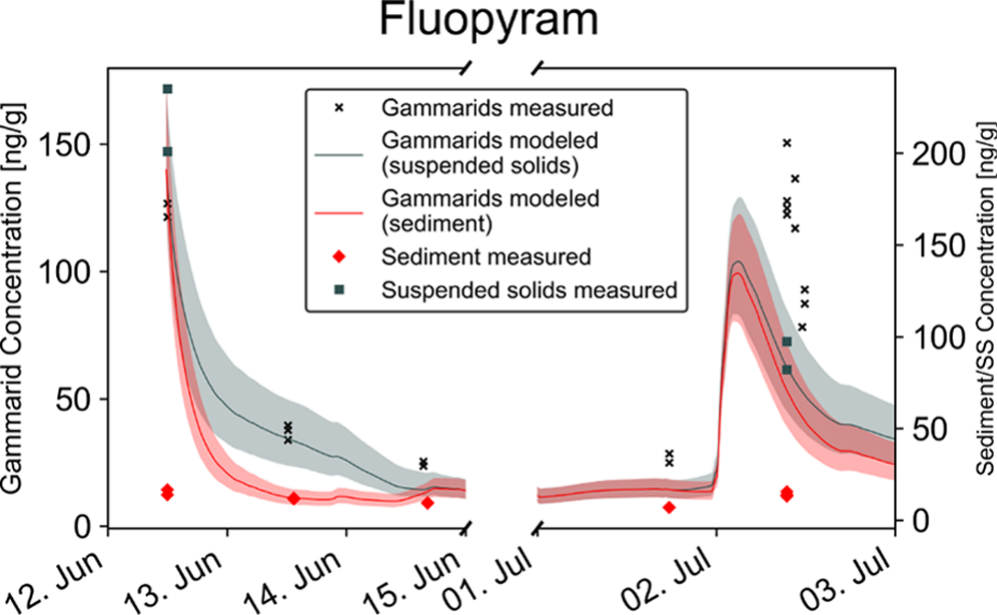
Modeled internal concentration of FLU when considering all uptake pathways and using suspended solid concentrations if available (gray) compared to uptake using sediment concentrations (red). Measured gammarid, sediment, and suspended solid concentrations are plotted for comparison.

The modeled pesticide concentrations increased by up to a factor of 9.5 when dietary uptake and sediment exposure were considered. However, a good fit between modeled and measured concentrations was only applicable to AZO and even there, the data points after the last rain events lay outside model uncertainty. For other pesticides, the additional pathways only have an impact greater than the model uncertainty some time after input events. There, the water concentration was reduced to baseline concentrations again, but the leaves and sediment concentrations were still elevated, resulting in the highest deviations between the model run with only aqueous uptake and the one including dietary uptake (see Figure 3 FLU June 13–15). Overall, the effect of dietary and sediment uptake was small, considering that all input parameters were chosen at the worst-case conditions (maximal assimilation efficiency and feeding rates). Subsequently, the hypothesis that the dietary uptake of contaminated leaves and/or sediment is responsible for the increased accumulation in the field is not supported by the field data. However, during rain events, the amount of suspended solids with higher pesticide concentrations increased strongly and sedimented in the cages. In order to determine the effect, the suspended solid concentrations were used as model input instead of the sediment concentrations. Under this condition, the model could explain the data points for FLU on June 13 (Figure 4), the day after the first suspended solid sample was taken, and partially explain those of CYP. While it improved the prediction for the second rain event (July 2), the measured concentrations were still outside the model uncertainty. This indicates, that suspended solids might be a more important pollution source for gammarids than detritus leaves or the sediment, which was not expected for such polar compounds. Unfortunately, suspended solid deposits in the cages were only visible after the two large rain events (June 12 and July 2) and no sufficiently large samples could be taken during low flow period, despite deployment of traps designed to deposit suspended solids. Thus, our data are not sufficient to answer whether the suspended solids are responsible for the increase in whole-body concentrations, but it looks like a promising direction for future studies. If it is shown in the future that the uptake from suspended solids is responsible for the discrepancy between lab and field data, including standardized organic particles with known contamination to laboratory tests used for authorization could improve their applicability to field conditions.

## 4 Conclusions

In conclusion, we could show that the pesticide pollution of aquatic invertebrates is highly dynamic. AZO, CYP, and FLU followed the concentration pattern in the water phase, while no depuration of THI by gammarids is evident. The low temporal resolution in sampling can explain part of the previously reported higher accumulation of pesticides in the field for aquatic invertebrates. However, even with very high temporal resolution, laboratory-derived TK data still underestimated the concentrations measured in the field systematically by up to a factor of 31 ± 3.0. We could show that even under worst case conditions, uptake from polluted detritus leaves and the sediment did not explain this underestimation but could be responsible for a minor proportion. Overall, several factors such as site-specific dependencies of TK rate constants or biochemical changes in the organisms due to the exposure might add up, but the systemic nature of the underestimation suggest that the primary driver of the slower TKs in the field is still unknown. Based on the observations discussed, uptake from suspended solids or slower depuration in the field, either due to lowered enzyme expression, mixture effects, or other biochemical changes might explain our results better, but further investigation is needed. Regardless of what the major contributor might be, the underestimation of bioaccumulation shows that laboratory-derived bioaccumulation data may not be protective enough and field studies should be used in order to retrospectively assess and possibly adapt the existing regulation process. For example, the underestimation ratios of a compound with similar properties could be used as a risk assessment factor to be more protective.

## Supporting Information Available

Details on field samples, preparation of artificial pond water, online SPE LC-HRMS/MS, target screening, analytical performance, and experimental determination of TK parameters (PDF)
