# Supplementary material for: Systematic Underestimation of Pesticide Burden for Invertebrates under Field Conditions: Comparing the Influence of Dietary Uptake and Aquatic Exposure Dynamics
Source: ACS Environ Au. 2021 Dec 9;2(2):166–75. doi: 10.1021/acsenvironau.1c00023 (PMC10114668; doi:10.1021/acsenvironau.1c00023)
Supplement: Supplementary file 1 — vg1c00023_si_002.pdf [file vg1c00023_si_002.pdf]

Supporting Information: Systematic Underestimation of Pesticide Burden for Invertebrates under Field Conditions: Comparing the Influence of Dietary Uptake to Aquatic Exposure Dynamics

Benedikt B. Lauper,[†,‡] Eva Anthamatten,[†,‡] Johannes Raths,[†,‡] Maricor Arlos,[†,¶] and Juliane Hollender[*,†,‡]

†*Department of Environmental Chemistry, Eawag, 8600 Dübendorf, Switzerland*

‡*Institute of Biogeochemistry and Pollutant Dynamics, ETH Zürich, 8092 Zürich, Switzerland*

¶*Department of Civil and Environmental Engineering, University of Alberta, 9211-116 St. NW, Edmonton, AB T6G 1H9, Canada*

E-mail: Juliane.Hollender@eawag.ch

Contents

1 Details on Field Samples **1**

1.1 Sampling Times 1

1.2 Water Temperature of Study Stream 3

2 Preparation of Artificial Pond Water (APW) **4**

3 Details on online SPE LC-HRMS/MS **4**

4 Target Screening **5**

4.1 Analytical Recoveries 5

4.2 Detected Pesticides Concentrations 7

5 Experimental Determination of TK Parameters **13**

5.1 Methods 13

5.2 Results 13

6 Toxicokinetic Modelling **15**

6.1 Error Propagation 15

6.2 Modelling of Pesticides with unknown TK Parameters 15

6.2.1 Model Parametrization 15

6.2.2 Model Results 17

6.3 Example Sagemath/Python code 20

6.3.1 Code Aqueous Uptake Model 20

6.3.2 Code Changes for Additional Uptake Pathways 35

7 Bibliography **37**

1 Details on Field Samples

1.1 Sampling Times

Table S1: Time of collection from source stream and sampling time for all gammarid samples. Each field sample shown resulted in duplicate samples for chemical analysis.

Sample Name	Time of Collection from Source Stream	Sampling Time
GAM Source Control 1	04.06.19 11:15	04.06.19 11:15
GAM Source Control 2	22.06.19 09:50	22.06.19 09:50
GAM Source Control 3	25.06.19 10:05	25.06.19 10:05
GAM Lab Control 1	04.06.19 11:15	12.06.19 17:30
GAM Lab Control 2	22.06.19 09:50	25.06.19 15:51
GAM Lab Control 3	25.06.19 10:05	02.07.19 16:01
GAM Study 1	04.06.19 11:15	12.06.19 12:00
GAM Study 2	04.06.19 11:15	13.06.19 12:13
GAM Study 3	04.06.19 11:15	14.06.19 15:29
GAM Study 4	04.06.19 11:15	15.06.19 12:21
GAM Study 5	04.06.19 11:15	16.06.19 10:15
GAM Study 6	04.06.19 11:15	16.06.19 18:22
GAM Study 7	04.06.19 11:15	17.06.19 11:55
GAM Study 8	04.06.19 11:15	18.06.19 12:00
GAM Study 9	04.06.19 11:15	19.06.19 11:15
GAM Study 10	04.06.19 11:15	20.06.19 11:30
GAM Study 11	04.06.19 11:15	20.06.19 13:50
GAM Study 12	04.06.19 11:15	21.06.19 09:57
GAM Study 13	04.06.19 11:15	22.06.19 11:38
GAM Study 14	04.06.19 11:15	22.06.19 17:02
GAM Study 15	04.06.19 11:15	23.06.19 10:53
GAM Study 16	04.06.19 11:15	24.06.19 11:20
GAM Study 17	22.06.19 09:50	25.06.19 11:00
GAM Study 18	22.06.19 09:50	01.07.19 17:42
GAM Study 19	22.06.19 09:50	02.07.19 09:28
GAM Study 20 [1)]	22.06.19 09:50	02.07.19 09:28
GAM Study 21	25.06.19 10:05	02.07.19 10:33
GAM Study 22 [2)]	25.06.19 10:05	02.07.19 11:30
GAM Study 23	25.06.19 10:05	02.07.19 11:50

[1] Duplicate field sample was used for spike tests to determine recoveries.

[2] Damaged cage resulted in only enough gammarids for one sample.

Table S2: Time of collection for all leaf, sediment and suspended solid concentration. Each field sample shown resulted in duplicate samples for chemical analysis.

Leaf samples	Time collected	Sediment Samples	Time collected	Suspended Solid Samples	Time collected
L Study 1	12.06.19 11:45	Sed Study 1	12.06.19 11:45	SuSo Study 1	12.06.19 11:45
L Study 2	13.06.19 11:27	Sed Study 2	13.06.19 13:24	SuSo Study 2	02.07.19 09:28
L Study 3	14.06.19 14:30	Sed Study 3	14.06.19 16:20		
L Study 4	15.06.19 12:21	Sed Study 4	15.06.19 12:21		
L Study 5	16.06.19 10:15	Sed Study 5	16.06.19 10:15		
L Study 6	16.06.19 18:22	Sed Study 6	16.06.19 18:22		
L Study 7	17.06.19 11:55	Sed Study 7	17.06.19 11:55		
L Study 8	18.06.19 12:00	Sed Study 8	18.06.19 12:00		
L Study 9	19.06.19 11:15	Sed Study 9	19.06.19 11:15		
L Study 10	20.06.19 11:30	Sed Study 10	20.06.19 11:30		
L Study 11	20.06.19 13:50	Sed Study 11	20.06.19 13:50		
L Study 12	21.06.19 09:57	Sed Study 12	21.06.19 09:57		
L Study 13	22.06.19 11:38	Sed Study 13	22.06.19 11:38		
L Study 14	22.06.19 17:02	Sed Study 14	22.06.19 17:02		
L Study 15	23.06.19 10:53	Sed Study 15	23.06.19 10:53		
L Study 16	24.06.19 11:20	Sed Study 16	24.06.19 11:20		
L Study 17	25.06.19 11:00	Sed Study 17	25.06.19 11:00		
L Study 18	01.07.19 17:42	Sed Study 18	01.07.19 17:42		
L Study 19	02.07.19 09:28	Sed Study 19	02.07.19 09:28		
L Study 20 [1)]	02.07.19 09:28	Sed Study 20 [1)]	02.07.19 09:28		

[1]Duplicate field sample was used for spike tests to determine recoveries.

1.2 Water Temperature of Study Stream

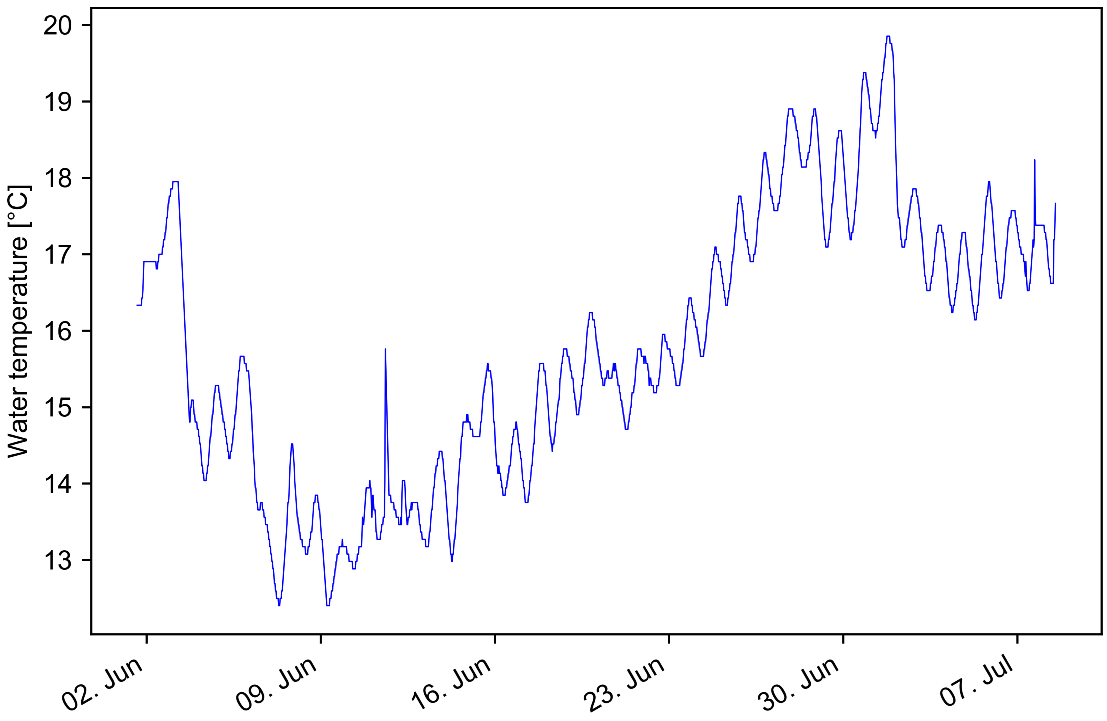

Figure S1: Water temperature of the study stream during the field study.

2 Preparation of Artificial Pond Water (APW)

APW was created according to Naylor et al[S1]. Calcium chloride (CaCl2; 158.0 mg), magnesium sulphate (MgSO4 · 7H2O; 480.5 mg), sodium hydrogen carbonate (NaHCO3; 258.9 g), and potassium chloride (KCl; 24 mg) were dissolved in 4 L of nanopure water. The solution was mixed thoroughly and ensured that a pH of 7.9 ± 0.3 was achieved by adjusting with concentrated sodium hydroxide or hydrogen chloride.

3 Details on online SPE LC-HRMS/MS

LCMS/MS analysis was carried out using a slightly modified version as published by Munz et al[S2]. and originally reported by Jeon et al[S3]. Online SPE cartridges were manually prepared by filling them in direction of flow with 9 mg Oasis HLB, and 9 mg an ion exchanger mix of Strata X-AX (33um), Strata X-CW (25 um, both Phenomex, Brechbühler AG, Switzerland) and Isolute ENV+ (70 um, Biotage, Sweden) in a ratio of 1:1:1.5. The HPLC system consisted of a tri-directional PAL autosampler (CTC Analytics, Switzerland), a 20 mL loop, a dispenser syringe, a low volume mixing chamber (Portmann Instruments AG), a high pressure gradient pump (Ultimate 3000 RS, Thermo Fischer Scientific) for the sample loading, a quaternary low-pressure mixing gradient pump (Rheos 2200, Flux instruments) for the SPE and an isocratic pump (Rheos 2000, Flux instruments) for the water gradient. 200 μL of organic sample extract was diluted by NPW up to a total volume of 20 mL. It was subsequently loaded into the 20 mL via dual injection (2 x 10 mL) with a flow rate of 2 mL/min using a loading solution of nanopure water containing 2mM ammonium acetate. The cartridge was later eluted using methanol (+ 0.1% formic acid) in back-flush mode at a flow rate of 40 l/min. The resulting eluate was mixed to initial LC conditions in a mixing chamber using nanopure water(+ 0.1% formic acid). Between samples the SPE cartridge and the loading loop were rinsed with acetonitrile and reconditioned with loading solution to prevent cross contamination.

Chromatographic separation was achieved for all sample types using a C18 column (Atlantis T3, 5 μm, 30 x 150 mm, Waters) and a mobile phase gradient of a water(A)/methanol(B) mixture (both with 0.1% formic acid). The gradient started at an 85:15 (A:B) ratio which was kept until minute 5. Subsequently it was linearly decreased to 5:95 at minute 20 where it was held until minute 29.5. Finally, the ratio was linearly increased back to 85:15 at minute 35. Over the whole run the flow rate and the column temperature were kept constant at 300 μl/min and 30 °C, respectively.

Ionization was achieved by electrospray ionization (ESI) source run in positive ionization mode with a spray voltage of +4 kV. The ion transfer capillary was heated to 320 °C. Full scan acquisition was performed for a m/z range 100-1000 at a resolution of 140'000 (at m/z 200) followed by top5 data dependent MS/MS using an inclusion list with all target compounds as well as most commonly applied pesticides in Switzerland.

4 Target Screening

4.1 Analytical Recoveries

Relative recoveries of the pesticide analytes were between 70 and 130% in all three matrices (table S3), apart from two compounds in the gammarids, four in the leaves and three in the sediment which showed higher deviations. Furthermore, three of the target compounds could not be recovered in gammarids and leaves (chlorpyrifos, chlorpyrifos-methyl, and pendimethalin) and three compounds could not be recovered in sediments (flonicamid, haloxyfop, and propamocarb). Since the compounds with relative recoveries outside the range were not detected in the study stream gammarids and only at low concentrations in the other matrices, no additional method development was completed to improve the analytical detections. Myclobutalin was excluded from analysis since a strong matrix interference made quantification unreliable.

Table S3: Relative recoveries for the screened pesticides in the water, gammarids, leaves and sediment. Chlorpyrifos, chlorpyrifos-methyl, myclobutanil and pendimethalin are not shown due to the analytical problems described above. (F) = fungicide, (H) = Herbicide and (I)= insecticide.

Pesticide (Type)	Gammarids [%]	Leaves [%]	Sediment [%]
Atrazin (H)	106	88	97
Azoxystrobin (F)	105	101	109
Boscalid (F)	102	116	90
Bupirimate (F)	107	116	107
Chloridazon (H)	102	73	94
Chlortoluron (H)	108	100	113
Clomazon (H)	97	170	70
Clothianidin (I)	96	98	102
Cyproconazole (F)	98	99	90
Cyprodinil (F)	104	92	110
Diazinon (I)	102	96	96
Difenoconazole (F)	115	68	167
Dimethachlor (H)	115	91	98
Dimethenamide (H)	107	85	103
Dimethoat (I)	112	93	103
Epoxiconazole (F)	95	108	103
Ethofumesat (H)	81	27	59
Fenhexamide (F)	105	102	103
Fenpyrazamine (F)	109	156	86
Flonicamide (I)	24	55	0
Fluopyram (F)	102	105	98
Haloxyfop (H)	104	96	0
Imidacloprid (I)	100	87	95
Iprovalicarb (F)	114	82	100
Isoproturon (H)	79	87	92
Lenacil (H)	101	95	100
Linuron (H)	108	104	96
Metamitron (H)	104	96	112
Metconazole (F)	104	86	98
Methoxyfenozid (I)	104	94	98
Metolachlor (H)	103	90	94
Metribuzin (H)	99	97	101
Napropamide (H)	119	107	181
Penconazole (F)	90	103	79
Pirimicarb (I)	117	84	109
Propamocarb (F)	113	88	0
Propiconazole (F)	100	98	100
Prosulfocarb (H)	61	14	71
Simazin (H)	106	99	101
Tebuconazole (F)	105	89	90
Tebufenozide (I)	115	94	100
Terbuthylazin (H)	103	92	97
Thiacloprid (I)	103	94	111
Thiamethoxam (I)	105	90	103
Trifloxystrobin (F)	110	30	81

4.2 Detected Pesticides Concentrations

Table S4: Maximal and minimal pesticide concentrations in the gammarids and water samples. (F) = fungicide; (H) = herbicide; (I) = insecticide. n.d= not detected; <LOQ = detected but not quantifiable; n.m = not measured.

Pesticide (Type)	Gammarids				Water			
	LOQ [ng/g_{ww}]	Max conc [ng/g_{ww}]	Min conc [ng/g_{ww}]	t(c_{max})	LOQ [ng/L]	Max conc [ng/L][1)]	Min conc [ng/L][1)]	t(c_{max})
Atrazin (H)	0.62	n.d	n.d	-	8	n.d	n.d	
Azoxystrobin (F)	0.62	40 ± 7.5	0.95 ± 0.090	02.07.19 11.50	5	6300 ± 440	n.d	02.07.19 00:22
Boscalid (F)	3.7	n.d	n.d	-	10	n.d	n.d	
Bupirimate (F)	2.5	2.9 ± 0.92	n.d	12.06.19 12.00	5	n.d	n.d	
Chloridazon (H)	0.12	n.d	n.d	-	15	n.d	n.d	
Chlortoluron (H)	0.76	n.d	n.d	-	20	n.d	n.d	
Clomazon (H)	0.81	n.d	n.d	-	n.m	n.m	n.m	
Clothianidin (I)	0.73	0.52 ± 0.18	n.d	19.06.19 11.15	15	38 ± 1.5	n.d	22.06.19 00:59
Cyproconazole (F)	0.76	n.d	n.d	-	35	n.d	n.d	
Cyprodinil (F)	0.62	54 ± 4.2	1.1 ± 0.22	02.07.19 09.28	10	720 ± 80	n.d	02.07.19 00:42
Diazinon (I)	0.19	n.d	n.d	-	10	n.d	n.d	
Difenoconazole (F)	2.5	2.5 ± 0.44	n.d	02.07.19 10.33	65	230 [2)]	n.d	19.06.19 15:16
Dimethachlor (H)	0.62	n.d	n.d	-	n.m	n.m	n.m	
Dimethenamide (H)	0.29	<LOQ	n.d	12.06.19 12.00	15	75 ± 2.3	n.d	12.06.19 15:23

Pesticide (Type)	Gammarids				Water			
	LOQ [ng/g_{ww}]	Max conc [ng/g_{ww}]	Min conc [ng/g_{ww}]	t(c_{max})	LOQ [ng/L]	Max conc [ng/L]	Min conc [ng/L]	t(c_{max})
Dimethoat (I)	0.62	n.d	n.d	-	15	n.d	n.d	12.06.19 14:43
Epoxiconazole (F)	1.5	<LOQ	n.d	12.06.19 12.00	15	52 ± 2.6	n.d	01.07.19 23:42
Ethofumesat (H)	8.60	n.d	n.d	-	40	n.d	n.d	
Fenhexamide (F)	1.3	17 ± 1.8	n.d	02.07.19 11.50	4	7600 ± 530	n.d	02.07.19 00:42
Fenpyrazamine (F)	0.62	11 ± 0.58	0.63 ± 0.066	16.06.19 10.15	15	4100 ± 210	20 ± 1.0	16.06.19 02:04
Flonicamide (I)	1.5	n.d	n.d	-	n.m	n.m	n.m	
Fluopyram (F)	1.3	140 ± 28	20 ± 0.78	12.06.19 12.00	4	31000 ± 930	370 ± 11	02.07.19 00:22
Haloxyfop (H)	1.7	n.d	n.d	-	20	n.d	n.d	
Imidacloprid (I)	0.62	0.8 ± 0.074	n.d	19.06.19 11.15	20	n.d	n.d	
Iprovalicarb (F)	0.27	n.d	n.d	-	10	n.d	n.d	
Isoproturon (H)	0.25	n.d	n.d	-	10	n.d	n.d	
Lenacil (H)	0.82	n.d	n.d	-	20	n.d	n.d	
Linuron (H)	2.7	n.d	n.d	-	n.m	n.m	n.m	
Metamitron (H)	0.62	0.79 ± 0.024	n.d	12.06.19 12.00	5	600 ± 60	n.d	12.06.19 15:23
Metconazole (F)	0.86	n.d	n.d	-	n.m	n.m	n.m	
Methoxyfenozid (I)	0.62	n.d	n.d	-	15	140 [2)]	n.d	15.06.19 20:22
Metolachlor (H)	0.26	8.5 ± 2.9	n.d	12.06.19 12.00	4	350 ± 3.5	n.d	12.06.19 14:43
Metribuzin (H)	0.91	n.d	n.d	-	70	n.d	n.d	

Pesticide (Type)	Gammarids LOQ [ng/g_{ww}]	Gammarids Max conc [ng/g_{ww}]	Gammarids Min conc [ng/g_{ww}]	Gammarids t(c_{max})	Water LOQ [ng/L]	Water Max conc [ng/L]	Water Min conc [ng/L]	Water t(c_{max})
Napropamide (H)	0.29	47 ± 2.7	1.1 ± 0.23	22.06.19 17.02	7	5200 ± 260	n.d	22.06.19 13:04
Penconazole (F)	0.82	5.9 ± 1.1	<LOQ	12.06.19 12.00	15	47 [2)]	n.d	02.07.19 00:42
Pirimicarb (I)	0.25	n.d	n.d	-	n.q	n.q.	n.q	
Propamocarb (F)	0.62	n.d	n.d	-	20	n.d	n.d	
Propiconazole (F)	0.73	n.d	n.d	-	6	n.d	n.d	
Prosulfocarb (H)	1.5	n.d	n.d	-	n.m	n.m	n.m	
Simazin (H)	0.77	<LOQ	n.d	12.06.19 12.00	2	73 ± 3.7	n.d	01.07.19 23:42
Tebuconazole (F)	0.80	n.d	n.d	-	6	17 ± 1.0	n.d	19.06.19 15:16
Tebufenozide (I)	2.5	n.d	n.d	-	30	n.d	n.d	
Terbuthylazin (H)	0.29	3.3 ± 0.4	<LOQ	12.06.19 12.00	2	170 ± 15	3.0 ± 0.27	01.07.19 23:42
Thiacloprid (I)	2.5	44 ± 0.34	21 ± 3.7	18.06.19 12.00	8	700 ± 63	n.d	02.07.19 01:02
Thiamethoxam (I)	0.12	0.37 ± 0.089	n.d	24.06.19 11.20	100	n.d	n.d	
Trifloxystrobin (F)	0.26	<LOQ	n.d	12.06.19 12.00	n.q	n.q	n.q	

[1)] Measurement error of the MS2Field was calculated from absolute median relative % difference as reported in la Cecilia et al[S4].

[2)] Measurment error of the MS2Field could not be calculated since the concentration at low flow conditions were below LOQ. For more information see la Cecilia et al[S4].

Table S5: Maximal and minimal pesticide concentrations in the leaf and sediment samples. (F) = fungicide; (H) = herbicide; (I) = insecticide. n.d= not detected; <LOQ = detected but not quantifiable.

Pesticide (Type)	Leaves				Sediment			
	LOQ [ng/g_{dw}]	Max conc [ng/g_{dw}]	Min conc [ng/g_{dw}]	$t(c_{max})$	LOQ [pg/g_{dw}]	Max conc [pg/g_{dw}]	Min conc [pg/g_{dw}]	$t(c_{max})$
Atrazin (H)	0.045	0.57 ± 0.11	n.d	20.06.19 13.50	1.4	300 ± 29	120 ± 1.4	19.06.19 11.15
Azoxystrobin (F)	0.20	130 ± 31	11 ± 0.46	02.07.19 09.28	4.2	2400 ± 210	650 ± 3.7	02.07.19 09.28
Boscalid (F)	2.8	n.d	n.d		12.0	120 ± 36	40 ± 1.1	17.06.19 11.55
Bupirimate (F)	0.042	22 ± 1.0	1.5 ± 0.061	12.06.19 11.45	2.1	830 ± 140	180 ± 1.4	17.06.19 11.55
Chloridazon (H)	0.075	0.33 ± 0.013	n.d	14.06.19 14.30	2.1	50 ± 3.3	18 ± 0.28	25.06.19 11.00
Chlortoluron (H)	0.063	n.d	n.d		5.7	n.d	n.d	
Clomazon (H)	0.13	<LOQ	n.d		2.1	77 ± 70	n.d	25.06.19 11.00
Clothianidin (I)	0.230	1.0 ± 0.059	n.d	24.06.19 11.20	4.0	79 ± 5.1	18 ± 0.92	25.06.19 11.00
Cyproconazole (F)	0.29	n.d	n.d		2.1	3.5 ± 3.5	n.d	15.06.19 12.21
Cyprodinil (F)	0.087	130 ± 2.3	8.3 ± 0.2	12.06.19 11.45	85.0	3400 ± 540	1100 ± 32	17.06.19 11.55
Diazinon (I)	0.50	n.d	n.d		8.5	13 ± 5.3	<LOQ	17.06.19 11.55
Difenoconazole (F)	1.0	94 ± 5.3	30 ± 1.2	01.07.19 17.42	21.0	2000 ± 360	560 ± 21	17.06.19 11.55
Dimethachlor (H)	0.085	n.d	n.d		3.7	n.d	n.d	
Dimethenamide (H)	0.16	1.4 ± 0.11	n.d	13.06.19 11.27	4.5	44 ± 1.5	<LOQ	12.06.19 15.00
Dimethoat (I)	0.10	n.d	n.d		1.4	n.d	n.d	

Pesticide (Type)	Leaves				Sediment			
	LOQ $[ng/g_{dw}]$	Max conc $[ng/g_{dw}]$	Min conc $[ng/g_{dw}]$	$t(c_{max})$	LOQ $[pg/g_{dw}]$	Max conc $[pg/g_{dw}]$	Min conc $[pg/g_{dw}]$	$t(c_{max})$
Epoxiconazole (F)	0.27	7.5 ± 0.0052	2.9 ± 0.25	14.06.19 14.30	8.5	240 ± 40	91 ± 0.87	17.06.19 11.55
Ethofumesat (H)	0.39	5.6 ± 1.3	n.d	12.06.19 11.45	85.0	240 ± 41	n.d	23.06.19 10.53
Fenhexamide (F)	1.3	30 ± 8.8	n.d	02.07.19 09.28	17.0	930 ± 50	61 ± 4.7	02.07.19 09.28
Fenpyrazamine (F)	0.050	140 ± 7.6	9.1 ± 0.42	16.06.19 10.15	4.2	130 ± 210	330 ± 11	17.06.19 11.55
Flonicamide (I)	0.34	n.d	n.d		4.4	n.d	n.d	
Fluopyram (F)	0.059	840 ± 24	140 ± 0.32	12.06.19 11.45	21.0	21000 ± 4100	7000 ± 16	17.06.19 11.55
Haloxyfop (H)	5.2	n.d	n.d		4.2	n.d	n.d	
Imidacloprid (I)	0.18	2.3 ± 0.21	n.d	12.06.19 11.45	2.3	27 ± 6.5	5.6 ± 1.1	17.06.19 11.55
Iprovalicarb (F)	0.081	n.d	n.d		2.1	n.d	n.d	
Isoproturon (H)	0.10	n.d	n.d		2.1	13 ± 3.7	4.7 ± 0.12	25.06.19 11.00
Lenacil (H)	0.12	n.d	n.d		4.2	8.3 ± 14	n.d	02.07.19 09.28
Linuron (H)	1.2	n.d	n.d		16.0	82 ± 12	48 ± 3.0	17.06.19 11.55
Metamitron (H)	0.080	24 ± 1.7	0.23 ± 0.041	12.06.19 11.45	1.8	400 ± 5.2	49 ± 0.74	12.06.19 15.00
Metconazole (F)	0.50	0.61 ± 0.047	n.d	18.06.19 12.00	4.2	16 ± 2.7	<LOQ	17.06.19 11.55
Methoxyfenozid (I)	0.17	3.1 ± 0.052	0.63 ± 0.032	01.07.19 17.42	7.5	57 ± 3.7	19 ± 0.91	25.06.19 11.00
Metolachlor (H)	0.08	46 ± 1.8	0.69 ± 0.0017	12.06.19 11.45	8.5	390 ± 0.65	32 ± 1.3	12.06.19 15.00
Metribuzin (H)	0.057	0.31 ± 0.043	n.d	12.06.19 11.45	2.1	27 ± 1.1	5.9 ± 0.048	12.06.19 15.00
Napropamide (H)	0.020	140 ± 5.3	20 ± 0.11	14.06.19 14.30	4.2	2600 ± 333	680 ± 4.9	23.06.19 10.53

Pesticide (Type)	Leaves				Sediment			
	LOQ [ng/g_{dw}]	Max conc [ng/g_{dw}]	Min conc [ng/g_{dw}]	t(c_{max})	LOQ [pg/g_{dw}]	Max conc [pg/g_{dw}]	Min conc [pg/g_{dw}]	t(c_{max})
Penconazole (F)	0.11	10 ± 0.42	3.0 ± 0.16	12.06.19 11.45	4.2	280 ± 52	67 ± 3.8	17.06.19 11.55
Pirimicarb (I)	0.057	12 ± 0.22	0.093 ± 0.0079	16.06.19 10.15	15.0	60 ± 10	<LOQ	20.06.19 13.50
Propamocarb (F)	0.16	0.31 ± 0.31	n.d	18.06.19 12.00	2100.0	n.d	n.d	
Propiconazole (F)	0.25	n.d	n.d		4.2	100 ± 1.1	6.4 ± 1.5	12.06.19 15.00
Prosulfocarb (H)	0.69	1.3 ± 1.3	n.d	13.06.19 11.27	8.5	<LOQ	<LOQ	
Simazin (H)	0.056	1.6 ± 0.042	n.d	12.06.19 11.45	2.1	170 ± 17	77 ± 5.6	19.06.19 11.15
Tebuconazole (F)	0.25	1.7 ± 0.091	0.29 ± 0.29	12.06.19 11.45	4.2	42 ± 7.7	14 ± 0.68	17.06.19 11.55
Tebufenozide (I)	0.25	4.8 ± 0.34	1.3 ± 0.06	01.07.19 17.42	4.2	150 ± 4.2	n.d	17.06.19 11.55
Terbuthylazin (H)	0.056	17 ± 0.46	0.62 ± 0.038	12.06.19 11.45	8.5	180 ± 3.0	34 ± 0.2	12.06.19 15.00
Thiacloprid (I)	0.50	48 ± 0.31	4.2 ± 0.044	12.06.19 11.45	3.9	610 ± 180	86 ± 10	17.06.19 11.55
Thiamethoxam (I)	0.50	0.79 ± 0.0062	n.d	19.06.19 11.15	7.4	54 ± 4.0	<LOQ	25.06.19 11.00
Trifloxystrobin (F)	0.50	3.5 ± 0.14	n.d	13.06.19 11.27	8.5	58 ± 7.2	n.d	25.06.19 11.00

5 Experimental Determination of TK Parameters

5.1 Methods

Toxicokinetic parameters (rate constants and BCF_{kin}) were obtained from laboratory bioconcentration experiments with *G. pulex* following the previously described study designs[S5–S7]. Slight deviations were made regarding the feeding to avoid a bias from dietary exposure. During the exposure phase of one day gammarids were not fed, but received leaf litter from their origin habitat as food for the following two days of elimination phase. Gammarids were exposed all together at 11 °C in a 6 L aquarium. The exposure mix consisted of twelve environmental relevant organic pollutants (atenolol, azoxystrobin, benzotriazole, citalopram, carbamazepine, cyprodinil, diclofenac, fluopyram, sulfamethoxazole, tebuconazole, terbutryn, thiacloprid) with a nominal concentration of 50 μg/L each. Medium and tissue concentrations were determined by online SPE LC-HRMS/MS as described by Fu et al[S5]. Toxicokinetic modelling was performed using a first-order one compartment model in the BYOM 4.2 platform (`http://www.debtox.nl/about.html`) in Matlab R2019a[S8]. All parameters were fitted simultaneously.

5.2 Results

The obtained toxicokinetic parameters for azoxystrobin, cyprodinil, fluopyram and thiacloprid which were used in the present study are presented in Table S6. The corresponding plots are presented in Figure S2

Table S6: Toxicokinetic parameters of the selected compounds. The 95 % convidence intervals (CIs) are shown in brackets. The quality of the model fit is described by AIC and R^2.

Compound	k_{up} [L/kg·d]		k_{el} [d^{-1}]		AIC	R^2	BCF_{kin} [L/kg]
Azoxystrobin	28.0	[20.1, 38.6]	3.7	[2.7, 5.1]	193	0.43	7.6
Cyprodinil	518.0	[428.9, 625.3]	10.1	[8.5, 12.0]	212	0.95	51.5
Fluopyram	70.5	[61.4, 80.7]	9	[7.9, 10.2]	153	0.97	7.8
Thiacloprid	4.3	[3.6, 5.2]	0.7	[0.4, 1.0]	155	0.74	6.3

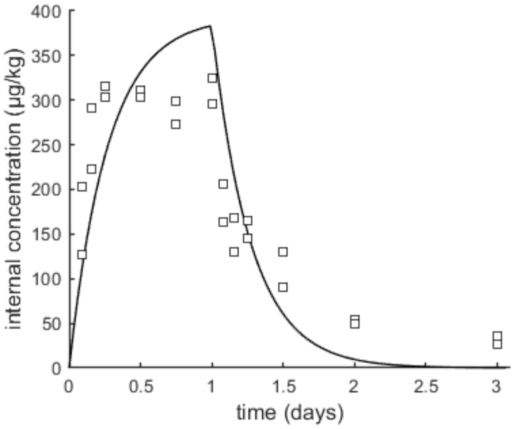

(a) Azoxystrobin

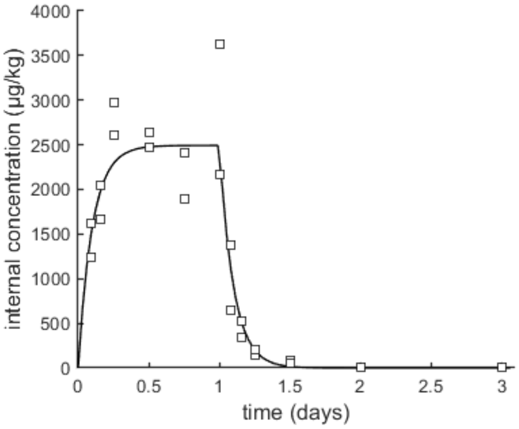

(b) Cyprodinil

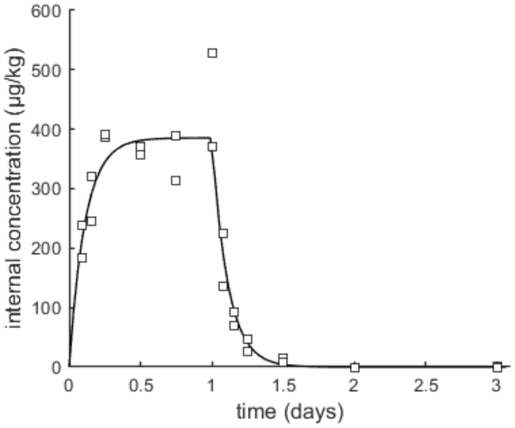

(c) Fluopyram

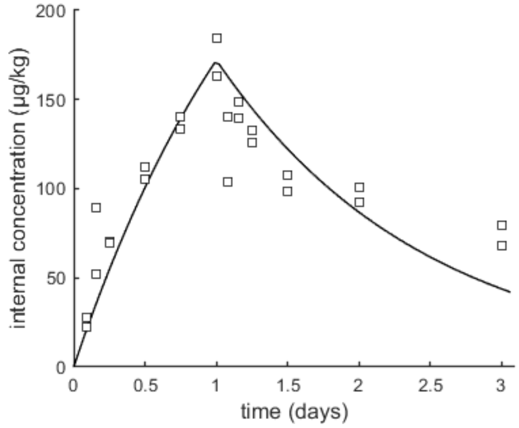

(d) Thiacloprid

Figure S2: Plotted TK models of the selected compounds: azoxystrobin (a), cyprodinil (b), fluopyram (c) and thiacloprid (d)

6 Toxicokinetic Modelling

6.1 Error Propagation

In the TK model upper and lower limits for predicted gammarids concentrations were calculated using error propagation: For upper limit calculation, the starting concentration $c_{org}(t = 0)$ was chosen based on the highest measured data point and the input concentrations, c_{leaf} and c_{sed} were interpolated linearly with 10'000 time-steps from the highest measured concentration at each time point. Subsequently, the model was run using the upper limit of the 95% confidence interval for the TK parameters influencing the pesticide uptake (k_{up}) and the lower 95% confidence interval limit for k_{el}, the only parameter influencing the pesticide depuration efficiency in the model. For the feeding rates fr_{leaf} and fr_{sed}, the means + standard deviations were used, since only standard deviations were available in literature and they were not convertible to 95 % CIs, since no sample numbers were given[S9,S10]. This results in the maximal possible accumulation of the pesticide within the 95% confidence interval for the aqueous uptake and slightly smaller error margins than the 95% CIs for the versions including dietary and sedimentary uptake. The lower limit was calculated accordingly but utilizing the minimal measured input concentrations and using the lower 95% confidence interval limits (or the mean - standard deviation for the feeding rates) for the uptake parameters and the upper limit for k_{el}.

6.2 Modelling of Pesticides with unknown TK Parameters

6.2.1 Model Parametrization

Table S7: Toxicokinetic constants and their source used for modelling of pesticides without own TK data. Values were chosen based on similarity of $\log D_{OW}$ values of the reference compounds to the pesticide in question. If TK data of multiple reference compounds within close $\log D_{OW}$ range were available, the best fitting was chosen.

Pesticide (Type)	k_u [L/kg·d]	k_{el} [d^{-1}]	Reference Compound	Reference Study
Fenhexamide	118.9 ± 16.56	8.464 ± 1.506	Diazinon	Ashauer et al. 2010[S11]
Fenpyrazamine	217 ± 20	6.66 ± 0.48	Epiconazole	Roesch et al 2016[S6]
Metolachlor	103.5 ± 9.552	4.559 ± 0.444	Propiconazole	Nyman et al. 2014[S12]
Napropamide	103.5 ± 9.552	4.559 ± 0.444	Propiconazole	Nyman et al. 2014[S12]
Penconazole	745 ± 124	3.91 ± 0.70	1,2,3-Trichlorobenzene	Ashauer et al. 2010[S13]

6.2.2 Model Results

When interpreting the results of the TK model calculated using TK constants of the reference compounds below, one has to be careful. While the reference compounds show similar distribution behaviour as the pesticides in question and the resulting model concentration fit the measured field concentration reasonably well, this could be coincidental and the correct TK parameters could deviate from the reference TK parameters strongly. In order to achieve more meaningful results, TK test as described in section 5 would need to be conducted.

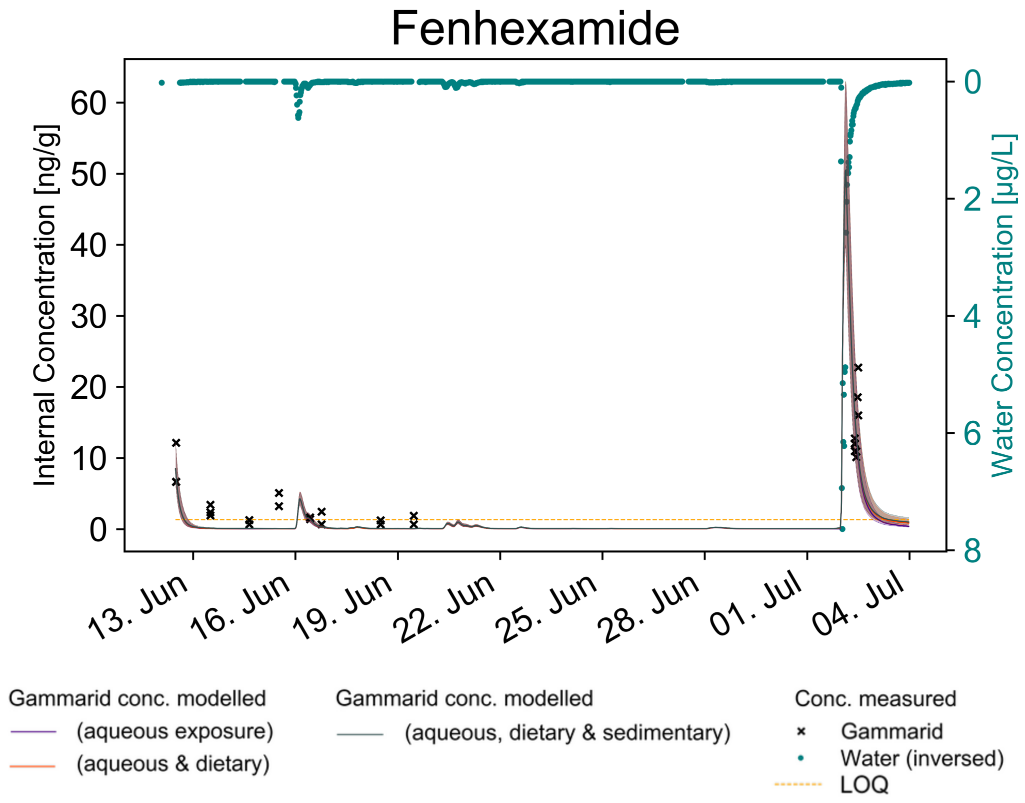

Figure S3: Modelled internal concentration of the pesticide fenhexamide in gammarids when considering only aqueous uptake (blue line), aqueous and dietary uptake combined (orange line) and when considering uptake from sediment as well (grey). Its measured values (black crosses), the LOQ (orange dotted line) and the measured water concentrations (indigo) are plotted for comparison.

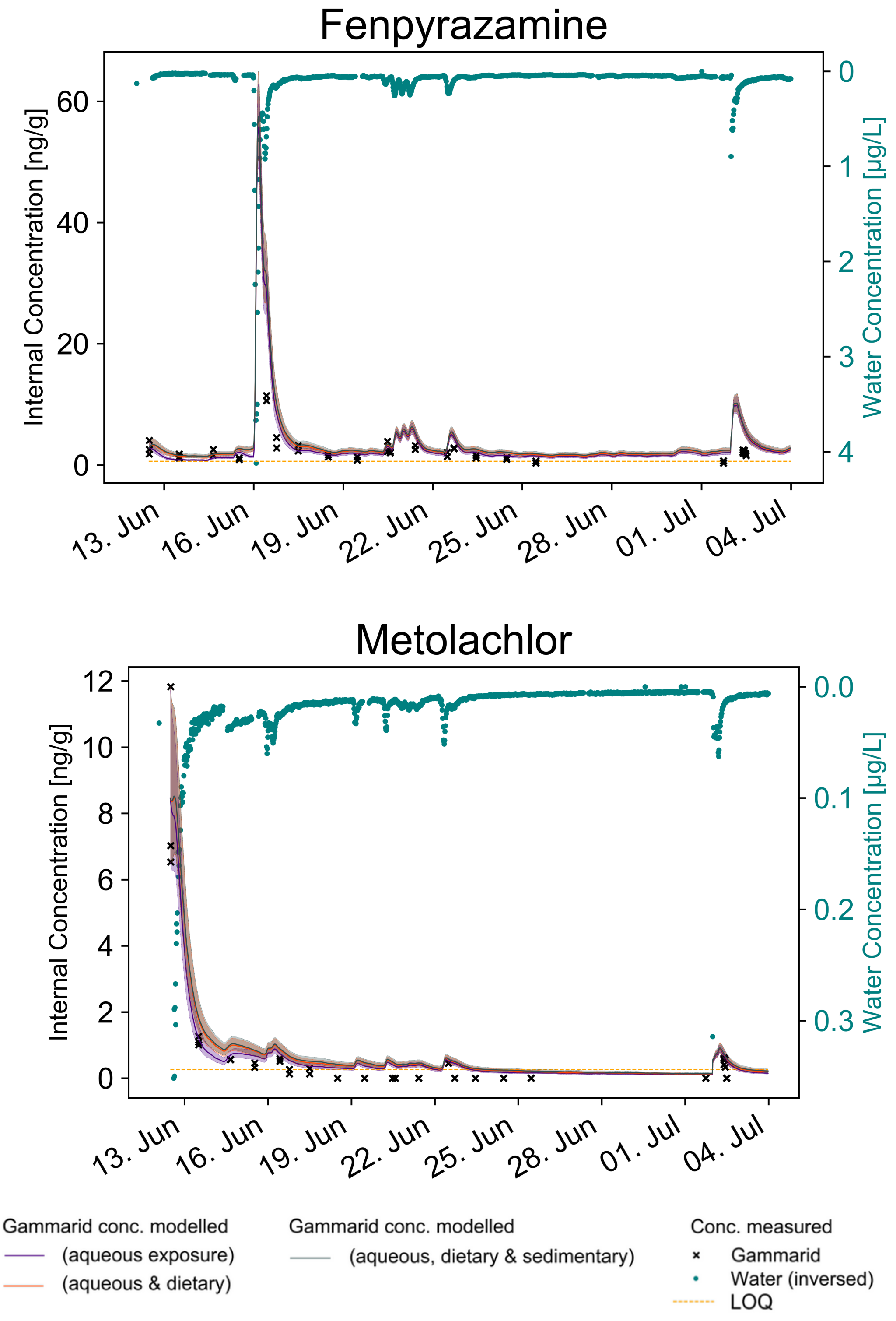

Figure S4: Modelled internal concentration of the pesticides fenpyrazamine and metolachlor in gammarids when considering only aqueous uptake (blue line), aqueous and dietary uptake combined (orange line) and when considering uptake from sediment as well (grey). Their measured values (black crosses), the LOQ (orange dotted line) and the measured water concentrations (indigo) are plotted for comparison.

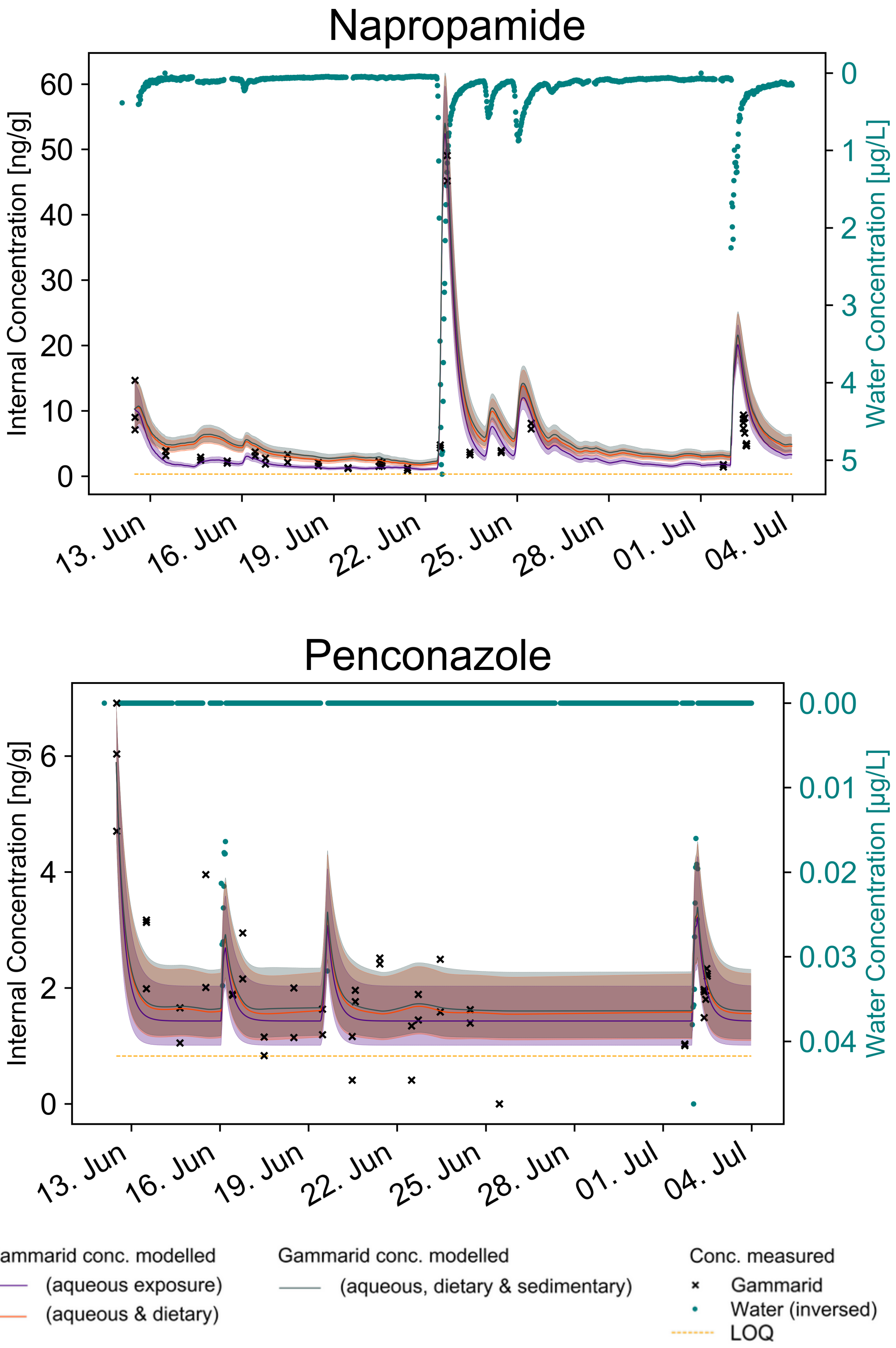

Figure S5: Modelled internal concentration of the pesticides napropamide and penconazole in gammarids when considering only aqueous uptake (blue line), aqueous and dietary uptake combined (orange line) and when considering uptake from sediment as well (grey). Their measured values (black crosses), the LOQ (orange dotted line) and the measured water concentrations (indigo) are plotted for comparison.

6.3 Example Sagemath/Python code

6.3.1 Code Aqueous Uptake Model

Code to model CYP uptake from only aqueous pathway. Changes to code to include all uptake pathways are shown in section 6.3.2.

```
#Import required Python-based open source software required for coding in SageMath
import csv
import numpy as np
import datetime
import matplotlib.pyplot as plt
from scipy.interpolate import interp1d
import os
import warnings
import pandas as pd

import matplotlib.dates as mdates
from matplotlib.dates import DateFormatter
from pandas.plotting import register_matplotlib_converters
register_matplotlib_converters()



###################################################################
#defining input for script --> Here are all parameters to be adapted
###################################################################

MP_name="Cyprodinil"
MP_name_short="Cypro"

dir_base=(r'C:\Users\lauperbe\TK_modeling\TK_Eschelisbach\0_linear_modeling\0_Vorlage')
```

```
#define input directory
dir_water_input=(r'C:\Users\lauperbe\TK_modeling\TK_Eschelisbach\0_linear_modeling\0_water_input\gammarid_test_period')
dir_gam_input=(r'C:\Users\lauperbe\TK_modeling\TK_Eschelisbach\0_linear_modeling\0_Gam_input')
dir_leaves_input=(r'C:\Users\lauperbe\TK_modeling\TK_Eschelisbach\0_linear_modeling\0_leaves_input')
dir_sediment_input=(r'C:\Users\lauperbe\TK_modeling\TK_Eschelisbach\0_linear_modeling\0_Sediment_input')

#define output directories
dir_output=os.path.join(dir_base,"1_output")
dir_output_aquatic=os.path.join(dir_output,"1_aquatic_only")
dir_output_combined=os.path.join(dir_output,"2_combined")
dir_quality_control_output=os.path.join(dir_output,'3_output_quality_control')
dir_waterdata_output=os.path.join(dir_output,'4_output_water_data')

#define input files
leaves_conc_file= MP_name_short+'_leaves_Esch.csv'
leaves_conc_file_no_extension=MP_name_short+'_leaves_Esch_no_extension.csv'
sediment_conc_file= MP_name_short+'_Sediment_Esch.csv'
sediment_conc_file_no_extension= MP_name_short+'_Sediment_Esch_no_extension.csv'
#water_conc_file='Azox_Waterconc_Esch_fictional_start_before.csv'
water_conc_file= MP_name_short+'_Waterconc_Esch_with_datapoint_before_error.csv'
GAM_conc_file=MP_name_short+'_GAM_measured_data.csv'
#both files need a data point at time 0 or before
#And interpolation time has to be shorter than time until last data point!

leaves_conc_type_used='conc_average' #define whether average, max or min value for leaves replicates are used
#for naming see columns leaves_conc_file
#df_leaves['conc_average'] uses average of replicates
#df_leaves['conc_min'] uses Minimal value of replicates
#df_leaves['conc_max'] uses Maximal value of replicates

sediment_conc_type_used='conc_average' #define whether average, max or min value for sediment replicates are used
#for naming see columns leaves_conc_file
#df_sediment['conc_average'] uses average of replicates
```

```
#df_sediment['conc_min'] uses Minimal value of replicates
#df_sediment['conc_max'] uses Maximal value of replicates

#There are 4 Files, all use the first leaves sample date as start time:
#Azox_Waterconc_Esch.csv: First water conc datapoint after error
#Azox_Waterconc_Esch_with_datapoint_before_error.csv: Includes last water conc before MS2Field error (has negative time to that datapoint)
#Azox_Waterconc_Esch_fictional_start_before.csv: Creats fictional data point at first leaves sample, uses conc before MS2Field error
#Azox_Waterconc_Esch_fictional_start_after.csv Creats fictional data point at first leaves sample, uses conc after MS2Field error
starting_date=datetime.datetime(2019,6,12,11,45,00)
end_date=datetime.datetime(2019,7,3,23,42,0)

######################################
#TK data Cyprodinil Johannes
######################################
ku= 518.00 #uptake rate constant, 1 L/kg-d
#ku_std= #standard deviation of uptake rate constant
ku_CI_up=625.30
ku_CI_down=428.9

kd= 10.05 #depuration rate constant, 1/d
#kd_std=1.35 #standard deviation of depuration rate constant
kd_CI_up=12.04
kd_CI_down=8.47
TK_data_suffix="_05_LOQ" #suffix to add to Plot names if TK data is manually adjusted
TK_label_suffix=", LOQ/2" #suffix to add to Plot legends names if TK data is manually adjusted

######################################
#food uptake parameters
######################################
#### uncontaminated control
ALPHA=1 #dimensionless - assimilation efficiency (random value at the moment)
```

```
k_feed= 0.128 #g_leaf(dry)/(g_gam(wet)*d) - feeding rate,
k_feed_stdev=0.009 #at the moment based on 95% confidence interval

Feed_suffix="" #suffix to add to Plot names if feed data is manually adjusted
Feed_label_suffix="" #suffix to add to Plot legends if feed data is manually adjusted
#should start with a spacer character

######################################
#sediment uptake parameters
######################################
#### Lopez
ALPHA_sed=1 #dimensionless - assimilation efficiency (random value at the moment)
k_sediment= 1.3 #g_sediment(dry)/(g_gam(wet)*d) - sediment feeding rate,
k_sediment_stdev=0.26 #based on 20 error assumption since no CI given!

sediment_suffix="" #suffix to add to Plot names if feed data is manually adjusted
sediment_label_suffix="" #suffix to add to Plot legends if feed data is manually adjusted
#should start with a spacer character

######################################
#time & interpolation data
######################################
#Define time in numerical approximation
t0=0 #starting time
tf=21.49 #end time, here I used the "end time" for my simulation in days
n=10000 #number of time steps, you can go one order of magnitude higher, but it will be slower
dt=(tf-t0)/(n-1) #size of your time steps in days
t=np.linspace(t0,tf,n) #populate time in your array

#dates_1=[datetime.datetime(2019,5,27)+datetime.timedelta(days=i) for i in t]

######################################
#Data handling
######################################
```

```
#Need to interpolate concentrations since resolution for numerical approximation is finer than the data that you
    ↪ have.
interpolation_type='linear' #type of interpolation used for interp1d function ('nearest','previous','cubic')
#is needed since time resolution of numerical approximation is better than data
#thus values between datta points have to be estimated
#--> see section below for evaluation

interpolation_type_leaves='linear'
interpolation_type_sediment='linear'

#handling of values below LOQ
#should c<LOQ be handeled as 0, LOQ/2 or LOQ
#in the first step all 3 version are calculated for aquatic only and compared -->choose from there
#(see plot MP_name_short+'_GAM_conc_min_LOQs_aquatic_only'+TK_data_suffix+Feed_suffix)

minimal_conc = "LOQ/2" # eigther "0", "LOQ/2" or LOQ
LOQ_choice="05_LOQ"

date_form = DateFormatter("%d. %b") #how dates should be displayed

Gam_data_plot_type="individual"
#"individual" plots each gammarid data point separately
#"average" plots

limit_calc = "advanced" #how to calculate upper/lower limits based on measured data (kinetic data is always
    ↪ treatet the same)
#options:
#"basic" : uses average concentration of replicate values, kinetic constants are only difference between upper/
    ↪ lower limits
#"advanced" : uses the highest/lowest replicate value for upper/lower limit resp.

limit_fill="TRUE" # should the space between upper and lower limit be filled with the colour?
#If "TRUE" it is filled; everything else it is left unfilled
```

```
draw_LOQ = "" #should the LOQ (Gam matrix) be drawn in the plots?
#eigther TRUE --> draws it; everything else: leaves it away

###automatic stuff

if limit_fill=="TRUE":
sediment_suffix=sediment_suffix+"_filled_limit"
dash_linewidth=0.1
else:
dash_linewidth=0.5

if draw_LOQ=="TRUE":
sediment_suffix=sediment_suffix+"_with_LOQ"
######################################
# Read your water data file
######################################
#change to input directory
os.chdir(dir_water_input)
#reading the data from your CSV file
df = pd.read_csv(water_conc_file,sep=',')
df.columns=['Counter','Filename','Date',MP_name,'LOQ','ConcMin0','ConcMin05LOQ','ConcMinLOQ','Starting_date','dt'
    ↪ ,'cum_d'] #name the columns of your dataframe
df.tail()#return the rows, visualize the data that you 'read' # this is just for visualization
df['Datetime']=pd.to_datetime(df.Date) #convert the dates from script to readable version in Python
df['Starting_date']=pd.to_datetime(df.Starting_date)

#Take measurment conc corresponding to minimal value handling
if minimal_conc=="0":
water_conc_measured=df.ConcMin0
print("minimal value = 0")

elif minimal_conc=="LOQ/2":
water_conc_measured=df.ConcMin05LOQ
print("minimal value = LOQ/2")
```

```
elif minimal_conc=="LOQ":
water_conc_measured=df.ConcMinLOQ
print("minimal value = LOQ")
else:
print("minimal concentration handling unknown")

######################################
#Read your leaves data file
######################################
#change to input directory
os.chdir(dir_leaves_input)
#reading the data from your CSV file
df_leaves = pd.read_csv(leaves_conc_file,sep=',')
df_leaves.columns=['Counter',"Compound",'Filename','Date','Starting_date','dt','cum_d',MP_name+'_sampleA',MP_name+'_sampleB',MP_name+'_sampleC','LOQ','conc_min','conc_max','conc_average','conc_stdev'] #name the columns of your dataframe
df_leaves.tail()#return the rows, visualize the data that you 'read' # this is just for visualization
df_leaves['Datetime']=pd.to_datetime(df_leaves.Date) #convert the dates from script to readable version in Python
df_leaves['Starting_date']=pd.to_datetime(df_leaves.Starting_date)


#load leaves data without extended datapoint
os.chdir(dir_leaves_input)
#reading the data from your CSV file
df_leaves_no_extension = pd.read_csv(leaves_conc_file_no_extension,sep=',')
df_leaves_no_extension.columns=['Counter',"Compound",'Filename','Date','Starting_date','dt','cum_d',MP_name+'_sampleA',MP_name+'_sampleB',MP_name+'_sampleC','LOQ','conc_min','conc_max','conc_average','conc_stdev'] #name the columns of your dataframe
df_leaves_no_extension.tail()#return the rows, visualize the data that you 'read' # this is just for visualization
df_leaves_no_extension['Datetime']=pd.to_datetime(df_leaves_no_extension.Date) #convert the dates from script to readable version in Python
df_leaves_no_extension['Starting_date']=pd.to_datetime(df_leaves_no_extension.Starting_date)
```

```
df_leaves_no_extension

#######################################
#Read your sediment data file
#######################################
os.chdir(dir_sediment_input)
#reading the data from your CSV file
df_sediment = pd.read_csv(sediment_conc_file,sep=',')
df_sediment.columns=['Counter',"Compound",'Filename','Date','Starting_date','dt','cum_d',MP_name+'_sampleA',
    ↪ MP_name+'_sampleB',MP_name+'_sampleC',MP_name+'_sampleD','LOQ','conc_min','conc_max','conc_average','
    ↪ conc_stdev'] #name the columns of your dataframe
df_sediment.tail()#return the rows, visualize the data that you 'read' # this is just for visualization
df_sediment['Datetime']=pd.to_datetime(df_sediment.Date) #convert the dates from script to readable version in
    ↪ Python
df_sediment['Starting_date']=pd.to_datetime(df_sediment.Starting_date)
df_sediment

#load sediment data without extended datapoint
os.chdir(dir_sediment_input)
#reading the data from your CSV file
df_sediment_no_extension = pd.read_csv(sediment_conc_file_no_extension,sep=',')
df_sediment_no_extension.columns=['Counter',"Compound",'Filename','Date','Starting_date','dt','cum_d',MP_name+'
    ↪ _sampleA',MP_name+'_sampleB',MP_name+'_sampleC',MP_name+'_sampleD','LOQ','conc_min','conc_max','
    ↪ conc_average','conc_stdev'] #name the columns of your dataframe
df_sediment_no_extension.tail()#return the rows, visualize the data that you 'read' # this is just for
    ↪ visualization
df_sediment_no_extension['Datetime']=pd.to_datetime(df_sediment_no_extension.Date) #convert the dates from script
    ↪  to readable version in Python
df_sediment_no_extension['Starting_date']=pd.to_datetime(df_sediment_no_extension.Starting_date)
df_sediment_no_extension

#######################################
#Read your GAM conc data file
#######################################
```

```
os.chdir(dir_gam_input)
#reading the data from your CSV file
df_GAM = pd.read_csv(GAM_conc_file,sep=',')
df_GAM.columns=['Counter',"Compound",'Filename','Date','Starting_date','dt','cum_d','MP_sampleA','MP_sampleB','MP_sampleC','LOQ','conc_min','conc_max','conc_average','conc_stdev'] #name the columns of your dataframe
df_GAM.tail()#return the rows, visualize the data that you 'read' # this is just for visualization
df_GAM['Datetime']=pd.to_datetime(df_GAM.Date) #convert the dates from script to readable version in Python
df_GAM['Starting_date']=pd.to_datetime(df_GAM.Starting_date)

#initial internal concentration concentration, ng/g
c_MP_int_start_average=df_GAM[['conc_average']].values[0][0] #average of internal concentration of 1st gammarid sampling timepoint (time 0) [ng/g]
c_MP_int_start_max=df_GAM[['conc_max']].values[0][0]
c_MP_int_start_min=df_GAM[['conc_min']].values[0][0]

LOQ_MP=df_GAM[['LOQ']].values[0][0]

######################################
#Read your water level data
######################################
dir_wl=r"C:\Users\lauperbe\TK_modeling\TK_Eschelisbach\0_linear_modeling\0_Rain_input\water_level_input"
file_wl="Esch_water_level_test_periode.csv"

os.chdir(dir_wl)
df_wl = pd.read_csv(file_wl,sep=',')
df_wl.columns=['Datetime',"Datum",'Zeit','waterlevel'] #name the columns of your dataframe
df_wl.tail()#return the rows, visualize the data that you 'read' # this is just for visualization
df_wl['Datetime']=pd.to_datetime(df_wl.Datetime) #convert the dates from script to readable version in Python

######################################
#Solving aquatic input only Ordinary Differential Equations for minimum conc=0
######################################
time_=df.cum_d #need the x_axis values (time) for interplotation
Cr=interp1d(time_,df.ConcMin0,kind=interpolation_type)
```

```
#need to create an array for your numerical solution, size should be the same as number of your timesteps
source=np.zeros([n])
c_MP_int_min0=np.zeros([n])

#initial internal concentration concentration, ng/g
c_MP_int_min0[0]=c_MP_int_start_average #could use control as starting concentration

for i in range (1,n):
source[i]=np.array(Cr(t[i])*ku/1000)
fslope1=np.array(source[i]-kd*c_MP_int_min0[i-1])
cg=np.array(c_MP_int_min0[i-1]+fslope1*dt)
fslope2=np.array(source[i]-kd*cg)
c_MP_int_min0[i]=np.array(c_MP_int_min0[i-1]+0.5*(fslope1+fslope2)*dt)

#######################################
#Solving aquatic input only Ordinary Differential Equations for minimum conc=LOQ/2
#######################################
time_=df.cum_d #need the x_axis values (time) for interplotation
Cr_Min05LOQ=interp1d(time_,df.ConcMin05LOQ,kind=interpolation_type)

#need to create an array for your numerical solution, size should be the same as number of your timesteps
source=np.zeros([n])
c_MP_int_min05_LOQ=np.zeros([n])

#initial internal concentration concentration, ng/g
c_MP_int_min05_LOQ[0]=c_MP_int_start_average

for i in range (1,n):
source[i]=np.array(Cr_Min05LOQ(t[i])*ku/1000)
fslope1=np.array(source[i]-kd*c_MP_int_min05_LOQ[i-1])
cg=np.array(c_MP_int_min05_LOQ[i-1]+fslope1*dt)
fslope2=np.array(source[i]-kd*cg)
c_MP_int_min05_LOQ[i]=np.array(c_MP_int_min05_LOQ[i-1]+0.5*(fslope1+fslope2)*dt)
```

```
########################################
#Solving aquatic input only Ordinary Differential Equations for minimum conc=LOQ
########################################
t=np.linspace(t0,tf,n) #populate time in your array

#Need to interpolate concentrations since resolution for numerical approximation is finer than the data that you
    ↪ have.
time_=df.cum_d #need the x_axis values (time) for interplotation
Cr_MinLOQ=interp1d(time_,df.ConcMinLOQ,kind=interpolation_type)

#need to create an array for your numerical solution, size should be the same as number of your timesteps
source=np.zeros([n])
c_MP_int_minLOQ=np.zeros([n])

#initial internal concentration concentration, ng/g
c_MP_int_minLOQ[0]=c_MP_int_start_average

for i in range (1,n):
source[i]=np.array(Cr_MinLOQ(t[i])*ku/1000)
fslope1=np.array(source[i]-kd*c_MP_int_minLOQ[i-1])
cg=np.array(c_MP_int_minLOQ[i-1]+fslope1*dt)
fslope2=np.array(source[i]-kd*cg)
c_MP_int_minLOQ[i]=np.array(c_MP_int_minLOQ[i-1]+0.5*(fslope1+fslope2)*dt)

########################################
#Solving aquatic input only Ordinary Differential Equations for chosen minimal concentration handling with TK
    ↪ data standard deviation Upper Limit
########################################

#choose internal concentration interpolated array
if minimal_conc=="0":
conc_interpol=Cr
```

```
elif minimal_conc=="LOQ/2":
conc_interpol=Cr_Min05LOQ

elif minimal_conc=="LOQ":
conc_interpol=Cr_MinLOQ
else:
print("minimal concentration handling unknown")

t=np.linspace(t0,tf,n) #populate time in your array

#need to create an array for your numerical solution, size should be the same as number of your timesteps
source=np.zeros([n])
c_MP_int_average=np.zeros([n]) #UL=upper Limit

#initial internal concentration concentration, ng/g
c_MP_int_average[0]=c_MP_int_start_average

for i in range (1,n):
source[i]=np.array(conc_interpol(t[i])*ku/1000)
fslope1=np.array(source[i]-kd*c_MP_int_average[i-1])
cg=np.array(c_MP_int_average[i-1]+fslope1*dt)
fslope2=np.array(source[i]-kd*cg)
c_MP_int_average[i]=np.array(c_MP_int_average[i-1]+0.5*(fslope1+fslope2)*dt)

###################################################
#HEUN METHOD for DIFFERENTIAL EQUATIONS UPPER Limit
###################################################

#Maximal accumulation: higest uptake & lowest depuration

ku_max=ku_CI_up
kd_min=kd_CI_down

#numerical method for solving differential equations, not as accurate as Rungga Kutta (RK), but not very slow
```

```
    ↪ either

t=np.linspace(t0,tf,n) #populate time in your array

#need to create an array for your numerical solution, size should be the same as number of your timesteps
source=np.zeros([n])
c_MP_int_UL=np.zeros([n]) #UL=upper Limit

#initial internal concentration concentration, ng/g
if limit_calc=="basic":
c_MP_int_UL[0]=c_MP_int_start_average
elif limit_calc=="advanced":
c_MP_int_UL[0]=c_MP_int_start_max
#with "basic" limit calculation the start conc is chosen as the average of all replicates
#with "advanced" limit calculation the start conc is chosen as the highest/lowest replicate respectively.

for i in range (1,n):
source[i]=np.array(conc_interpol(t[i])*ku_max/1000)
fslope1=np.array(source[i]-kd_min*c_MP_int_UL[i-1])
cg=np.array(c_MP_int_UL[i-1]+fslope1*dt)
fslope2=np.array(source[i]-kd_min*cg)
c_MP_int_UL[i]=np.array(c_MP_int_UL[i-1]+0.5*(fslope1+fslope2)*dt)

#####################################################
#HEUN METHOD for DIFFERENTIAL EQUATIONS LOWER Limit
#####################################################
#Minimal accumulation: lowest uptake & highest depuration
#k_up_min=k_up(average)-k_up(stdev)
#k_dep_max=k_dep(average)+k_dep(stdev)

#ku_min=ku-ku_std
#kd_max=kd+kd_std
ku_min=ku_CI_down
kd_max=kd_CI_up
```

```
#numerical method for solving differential equations, not as accurate as Rungga Kutta (RK), but not very slow either

t=np.linspace(t0,tf,n) #populate time in your array
#need to create an array for your numerical solution, size should be the same as number of your timesteps
source=np.zeros([n])
c_MP_int_LL=np.zeros([n])

#initial internal concentration concentration, ng/g
if limit_calc=="basic":
c_MP_int_LL[0]=c_MP_int_start_average #LL=lower limit
elif limit_calc=="advanced":
c_MP_int_LL[0]=c_MP_int_start_min
#with "basic" limit calculation the start conc is chosen as the average of all replicates
#with "advanced" limit calculation the start conc is chosen as the highest/lowest replicate respectively.

for i in range (1,n):
source[i]=np.array(conc_interpol(t[i])*ku_min/1000)
fslope1=np.array(source[i]-kd_max*c_MP_int_LL[i-1])
cg=np.array(c_MP_int_LL[i-1]+fslope1*dt)
fslope2=np.array(source[i]-kd_max*cg)
c_MP_int_LL[i]=np.array(c_MP_int_LL[i-1]+0.5*(fslope1+fslope2)*dt)

#WRITING RESULTS INTO CSV (upper vs lower Limit)
#Just need to convert your dates to date.time
increment=np.zeros([n])
#increment[0]=(tf-t0)/(n-1)
increment[0]=0
for i in range (1,n): #has to be from 1 otherwise it will set increment 0 to first difference,
#shifting the starting concentration to the 1st time point nummerically solved
dt=(tf-t0)/(n-1)
increment[i]=increment[i-1]+dt
```

```
#dates_1=[datetime.datetime(2019,5,27,14,33,56)+datetime.timedelta(days=i) for i in increment]
dates_1=[starting_date+datetime.timedelta(days=i) for i in increment]


solution = zip(dates_1,c_MP_int_average,c_MP_int_LL,c_MP_int_UL)

os.chdir(dir_output_aquatic)
writefile = csv.writer(open(aqu_only_out_filename, 'w'))


with open(aqu_only_out_filename, 'w') as f:
c = csv.writer(f)
header=['date',"internal conc "+MP_name_short+ " (ng/g)",'internal conc '+MP_name_short+' lower limit (ng/g)','
    ↪ internal conc '+MP_name_short+' upper limit (ng/g)']
c.writerow((header))
c.writerows(solution)
```

6.3.2 Code Changes for Additional Uptake Pathways

Code shown here needs input code from start of section 6.3.1, which was left away here to avoid duplication. Upper and lower limit calculations are done as shown in section 6.3.1.

```
#############################################################
#Solving the ODE with aquatic, dietary and sedimentary uptake
#############################################################
sediment_conc=df_sediment[sediment_conc_type_used] #choose which sediment conc to use
#options:
#df_sediment['conc_average'] uses average of replicates
#df_sediment['conc_min'] uses Minimal value of replicates
#df_sediment['conc_max'] uses Maximal value of replicates
leaves_conc=df_leaves[leaves_conc_type_used] #choose which leaves conc to use
#options:
#df_leaves['conc_average'] uses average of replicates
#df_leaves['conc_min'] uses Minimal value of replicates
#df_leaves['conc_max'] uses Maximal value of replicates

# i will interpolate the leaves and sediment concentration correspondlingly to the river concentration:
time_leaves=df_leaves.cum_d #need the x_axis values (time) for interplotation
C_leaves=interp1d(time_leaves,leaves_conc,kind=interpolation_type_leaves)
C_sediment=interp1d(time_leaves,sediment_conc,kind=interpolation_type_sediment)

###adapted Heun Method####
source=np.zeros([n])
c_MP_int_comb=np.zeros([n]) #still populating array first

#initial concentration, ng/g (first datapoint)
c_MP_int_comb[0]=c_MP_int_start_average #this will be first datapoint

for i in range (1,n):
       source[i]=np.array(conc_interpol(t[i])*ku/1000 + C_leaves(t[i])*ALPHA*k_feed+C_sediment(t[i])*ALPHA_sed*
          ↪ k_sediment) #conc_interpol is river conc interpolated
```

```
        fslope1=np.array(source[i]-kd*c_MP_int_comb[i-1])
        cg=np.array(c_MP_int_comb[i-1]+fslope1*dt)
        fslope2=np.array(source[i]-kd*cg)
        c_MP_int_comb[i]=np.array(c_MP_int_comb[i-1]+0.5*(fslope1+fslope2)*dt)
```

7 Bibliography

(S1) Naylor, C.; Maltby, L.; Calow, P. Scope for growth in Gammarus pulex, a freshwater benthic detritivore. *Hydrobiologia* **1989**, *188*, 517–523.

(S2) Munz, N. A.; Fu, Q.; Stamm, C.; Hollender, J. Internal Concentrations in Gammarids Reveal Increased Risk of Organic Micropollutants in Wastewater-Impacted Streams. *Environmental Science & Technology* **2018**, *52*, 10347–10358.

(S3) Jeon, J.; Kurth, D.; Ashauer, R.; Hollender, J. Comparative toxicokinetics of organic micropollutants in freshwater crustaceans. *Environmental Science & Technology* **2013**, *47*, 8809–8817.

(S4) la Cecilia, D.; Dax, A.; Ehmann, H.; Koster, M.; Singer, H.; Stamm, C. Continuous high-frequency pesticide monitoring to observe the unexpected and the overlooked. *Water Research X* **2021**, *13*, 100125.

(S5) Fu, Q.; Rösch, A.; Fedrizzi, D.; Vignet, C.; Hollender, J. Bioaccumulation, Biotransformation, and Synergistic Effects of Binary Fungicide Mixtures in Hyalella azteca and Gammarus pulex: How Different/Similar are the Two Species? *Environmental science & technology* **2018**, *52*, 13491–13500.

(S6) Rösch, A.; Anliker, S.; Hollender, J. How Biotransformation Influences Toxicokinetics of Azole Fungicides in the Aquatic Invertebrate Gammarus pulex. *Environmental Science & Technology* **2016**, *50*, 7175–7188.

(S7) Rösch, A.; Gottardi, M.; Vignet, C.; Cedergreen, N.; Hollender, J. Mechanistic Understanding of the Synergistic Potential of Azole Fungicides in the Aquatic Invertebrate Gammarus pulex. *Environmental Science & Technology* **2017**, *51*, 12784–12795.

(S8) MATLAB, *version 9.6 (R2019a)*; The MathWorks Inc.: Natick, Massachusetts, 2019.

(S9) Lopez, G.; Levinton, J. Ecology of Deposit-Feeding Animals in Marine Sediments. *Quarterly Review of Biology - QUART REV BIOL* **1987**, *62*, 235–260.

(S10) Maltby, L.; Clayton, S. A.; Wood, R. M.; McLoughlin, N. Evaluation of the Gammarus pulex in situ feeding assay as a biomonitor of water quality: Robustness, responsiveness, and relevance. *Environmental Toxicology and Chemistry* **2002**, *21*, 361–368.

(S11) Ashauer, R.; Hintermeister, A.; Caravatti, I.; Kretschmann, A.; Escher, B. I. Toxicokinetic and Toxicodynamic Modeling Explains Carry-over Toxicity from Exposure to Diazinon by Slow Organism Recovery. *Environmental Science & Technology* **2010**, *44*, 3963–3971.

(S12) Nyman, A.-M.; Schirmer, K.; Ashauer, R. Importance of Toxicokinetics for Interspecies Variation in Sensitivity to Chemicals. *Environmental Science & Technology* **2014**, *48*, 5946–5954.

(S13) Ashauer, R.; Caravatti, I.; Hintermeister, A.; Escher, B. I. Bioaccumulation kinetics of organic xenobiotic pollutants in the freshwater invertebrate Gammarus pulex modeled with prediction intervals. *Environmental Toxicology and Chemistry* **2010**, *29*, 1625–1636.
